# Transcriptome and proteome analysis of walnut (*Juglans regia* L.) fruit in response to infection by *Colletotrichum gloeosporioides*

**DOI:** 10.1186/s12870-021-03042-1

**Published:** 2021-05-31

**Authors:** Hongcheng Fang, Xia Liu, Yuhui Dong, Shan Feng, Rui Zhou, Changxi Wang, Xinmei Ma, Jianning Liu, Ke Qiang Yang

**Affiliations:** 1 College of Forestry, Shandong Agricultural University, Tai’an, Shandong Province China; 2 State Forestry and Grassland Administr, ation Key Laboratory of Silviculture inthe Downstream Areas of the Yellow River, Shandong Agricultural University, Tai’an, Shandong Province China; 3 Shandong Taishan Forest Ecosystem Research Station, Shandong Agricultural University, Tai’an, Shandong Province China; 4 Department of Science and Technology, Qingdao Agricultural University, Qingdao, Shandong Province China

**Keywords:** Walnut, *Colletotrichum gloeosporioides*, Transcriptomic, Proteomic, Differentially expressed genes (DEGs)

## Abstract

**Background:**

Walnut anthracnose induced by *Colletotrichum gloeosporioides* is a disastrous disease affecting walnut production. The resistance of walnut fruit to *C. gloeosporioides* is a highly complicated and genetically programmed process. However, the underlying mechanisms have not yet been elucidated.

**Results:**

To understand the molecular mechanism underlying the defense of walnut to *C. gloeosporioides*, we used RNA sequencing and label-free quantitation technologies to generate transcriptomic and proteomic profiles of tissues at various lifestyle transitions of *C. gloeosporioides*, including 0 hpi, pathological tissues at 24 hpi, 48 hpi, and 72 hpi, and distal uninoculated tissues at 120 hpi, in anthracnose-resistant F26 fruit bracts and anthracnose-susceptible F423 fruit bracts, which were defined through scanning electron microscopy. A total of 21,798 differentially expressed genes (DEGs) and 1929 differentially expressed proteins (DEPs) were identified in F26 vs. F423 at five time points, and the numbers of DEGs and DEPs were significantly higher in the early infection stage. Using pairwise comparisons and weighted gene co-expression network analysis of the transcriptome, we identified two modules significantly related to disease resistance and nine hub genes in the transcription expression gene networks. Gene Ontology and Kyoto Encyclopedia of Genes and Genomes analysis of the DEGs and DEPs revealed that many genes were mainly related to immune response, plant hormone signal transduction, and secondary metabolites, and many DEPs were involved in carbon metabolism and photosynthesis. Correlation analysis between the transcriptome data and proteome data also showed that the consistency of the differential expression of the mRNA and corresponding proteins was relatively higher in the early stage of infection.

**Conclusions:**

Collectively, these results help elucidate the molecular response of walnut fruit to *C. gloeosporioides* and provide a basis for the genetic improvement of walnut disease resistance.

**Supplementary Information:**

The online version contains supplementary material available at 10.1186/s12870-021-03042-1.

## Background

During the co-evolution of plant and pathogenic microorganisms, plants evolved a sophisticated innate immune system to defend against pathogen infection [1, 2]. The plant innate immune system mainly includes pathogen-associated molecular pattern (PAMP)-triggered immunity (PTI) and effector-triggered immunity (ETI), which rely on the MAPK cascade signaling network and molecular recognition to regulate the expression of corresponding genes, initiate a defense response, and produce systemic acquired resistance (SAR) through the salicylic acid (SA)-mediated signaling network [3, 4]. PTI is a basic defense response triggered by pattern-recognition receptors (PRRs), which detect conserved PAMPs [5–7]. Numerous proteins encoded by resistance genes (*R*) act as intracellular immune receptors to recognize pathogen effector proteins and activate ETI, which in turn typically induces the local hypersensitive response (HR) cell death to restrict pathogen growth and propagation [8, 9]. Most intracellular receptors are nucleotide-binding, leucine-rich repeat proteins (NLRs), which play different roles in plant immunity [10, 11]. To date, more than 213 typical *R* genes conferring resistance to multiple types of pathogens have been functionally identified in wheat (*Triticum aestivum* L.), rice (*Oryza sativa* L.), barley (*Hordeum vulgare* L.), maize (*Zea mays* L.), and other crop species. The local HR can also induce systemic acquired resistance (SAR) through the generation of mobile signals, accumulation of the defense hormone SA, and secretion of antimicrobial pathogenesis-related (PR) proteins.

A large number of genes, proteins, and metabolites participate in the process of plant innate immunity and cross talk with each other in a complex network system [2, 12]. With the continuous development of “omics” technology, networks and pathways have been reconstructed using transcriptome and proteome data. However, due to post-transcriptional, translational, or post-translational regulatory mechanisms and the complexity of alternative splicing, the transcriptional changes detected are not necessarily reflected in the expression of the corresponding determinative proteins [13–15]. Therefore, the integration of transcriptome and proteome data with various bioinformatics research methods can improve our understanding of the molecular networks that underlie biological processes [16]. Hub genes and key proteins operating in wheat-*Pst* (*Puccinia striiformis* f. sp. *tritici*) interactions were identified using pairwise comparisons and weighted gene co-expression network analysis (WGCNA) [13]. During soybean (*Glycine max*) symbiosis with arbuscular mycorrhizal fungi, key pathways and differentially expressed proteins (DEPs) were found to be involved in processes associated with “phenylalanine metabolism,” “plant hormone signal transduction,” “plant-pathogen interaction,” and “metabolic pathways” through Gene Ontology (GO) and Kyoto Encyclopedia of Genes and Genomes (KEGG) analysis [17]. The integration of transcriptome and metabolomic data sets greatly improves the predictive power of gene regulatory networks (GRNs) [18].

Walnut (*Juglans regia* L.), a diploid species (2n = 32) native to the mountainous regions of Central Asia, is cultivated worldwide for nut production [18]. Martínez-García et al. [19] published the reference genome of ‘Chandler’ (approximately 606 Mb per 1C genome), which enabled whole-genome resequencing [13, 20], the genetic dissection of important agronomical traits in walnut [21, 22], and the development of high-density genotyping tools [23]. Walnut anthracnose induced by *Colletotrichum gloeosporioides* (Penz.) Penz. and Sacc causes fruit gangrene and leaf scorching, resulting in up to 50% yield losses, and has been reported to be a serious issue in walnut production [24]. Most *Colletotrichum* species complete the pathogenic process in a hemibiotrophic manner. First, conidia germinate to generate appressoria that produce penetration pegs to begin initial infection. The penetration peg produces primary hyphae in the plant cells, secretes small molecular proteins, and absorbs secondary metabolites to enter biotrophy. Finally, the secondary hyphae generated by the primary hyphae grow rapidly in plant cells, release CAZymes, and disintegrate plant cells into necrotrophy [25–27]. Anthracnose is characterized by a long incubation period, concentrated onset time, and strong outbreak. The use of chemical fungicides is still the main method of disease control [28]. There are few reports on the molecular mechanism of resistance to *C. gloeosporioides* in walnut. An integrated proteomics and transcriptomics approach can be used to further understand the mechanism of resistance of walnut to *C. gloeosporioides*. In this paper, we observed the lifestyle transitions of *C. gloeosporioides* in the fruit bracts after inoculation. Label-free quantitation and Illumina RNA sequencing technology (RNA-seq) were combined to study the hub genes and proteins involved in walnut resistance to *C. gloeosporioides* at different time points. The results elucidated the molecular mechanisms of walnut resistance to *C. gloeosporioides* and can be used to facilitate walnut disease resistance breeding and intensive cultivation.

## Results

### Lifestyle transitions of *C. gloeosporioides* in infected walnut fruits

**Fig. 1:**
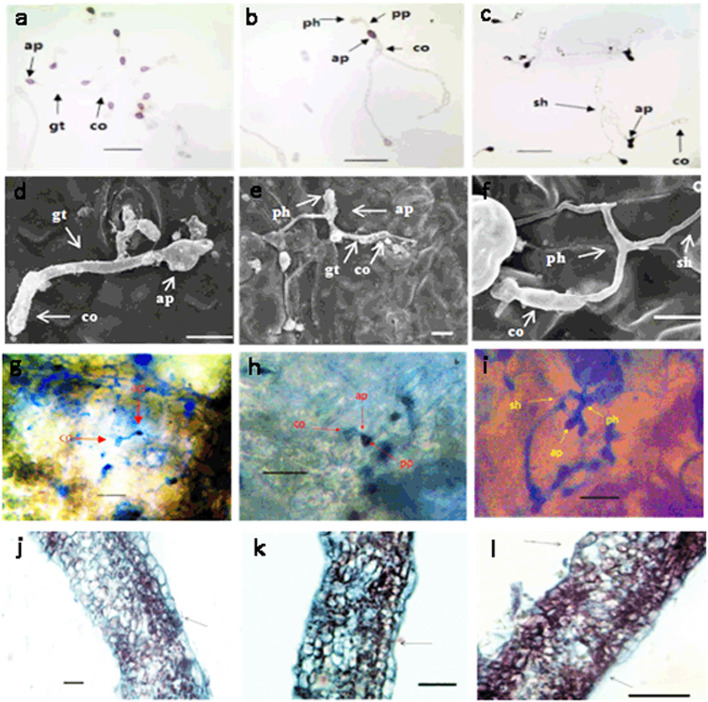
Lifestyle transitions of *C. gloeosporioides* conidia cultured in glucose solution and observed with light microscopy (**a**–**c**, **j**–**l**) or scanning electron microscopy (**d**–**f**). **a** and **d**, germinated conidia form melanized appressoria which attach to the surface of the host cell (24 hpi). **b** and **e**, the fungus then proliferates and appressoria form bulbous primary biotrophic hyphae (48 hpi). **c** and **f**, a thin filamentous secondary hyphae then forms, which is associated with the switch to necrotrophy (72 hpi). **g**–**l** Life cycle of *C. gloeosporioides* in infected intact leaves (**g**–**i**) and in paraffin section stained with aniline blue (**j**–**l**) at 24, 48 and 72 hpi. co, conidia; ap, appressoria; gt, germ tube; ph, primary hyphae; pp, penetration peg; sh, secondary hyphae. Bars: 50 μm (**a**–**c**), 10 μm (**d**–**f**), 100 μm (**g**–**l**)

To define the lifestyle transitions of *C. gloeosporioides* in infected walnut fruit bracts, the anatomical characteristics of the fruit bracts infected by *C. gloeosporioides* were observed using scanning electron microscopy, aniline blue staining, and paraffin sectioning. The results showed that *C. gloeosporioides* conidia germinated on fruit bracts and differentiated to appressoria within 6–12 hpi. The appressoria displayed a thin penetration peg at 24 hpi (Fig. 1a, d, g, j). The primary hyphae produced by the penetration peg appeared in the intracellular space at 48 hpi, which marks the beginning of biotrophy (Fig. 1b, e, h, k). The infection then entered a necrotrophic stage where the fungus formed secondary and necrotrophic hyphae at 72 hpi (Fig. 1c, f, i, l), and distal uninoculated tissues access to the resistance at 120 hpi. Thus, according to the lifestyle transitions of *C. gloeosporioides*, the resistant fruits (F26) and susceptible fruits (F423) were collected and inoculated according to the description by Feng et al. [29]. The fruit bract tissues of F26 and F423 at 0 hpi, pathological tissues at 24 hpi, 48 hpi, 72 hpi, and distal uninoculated tissues at 120 hpi were used for the transcriptome and proteome analysis.

### Comparative analysis of the response of F26 and F423 to *C. gloeosporioides* at the transcriptome level

**Fig. 2:**
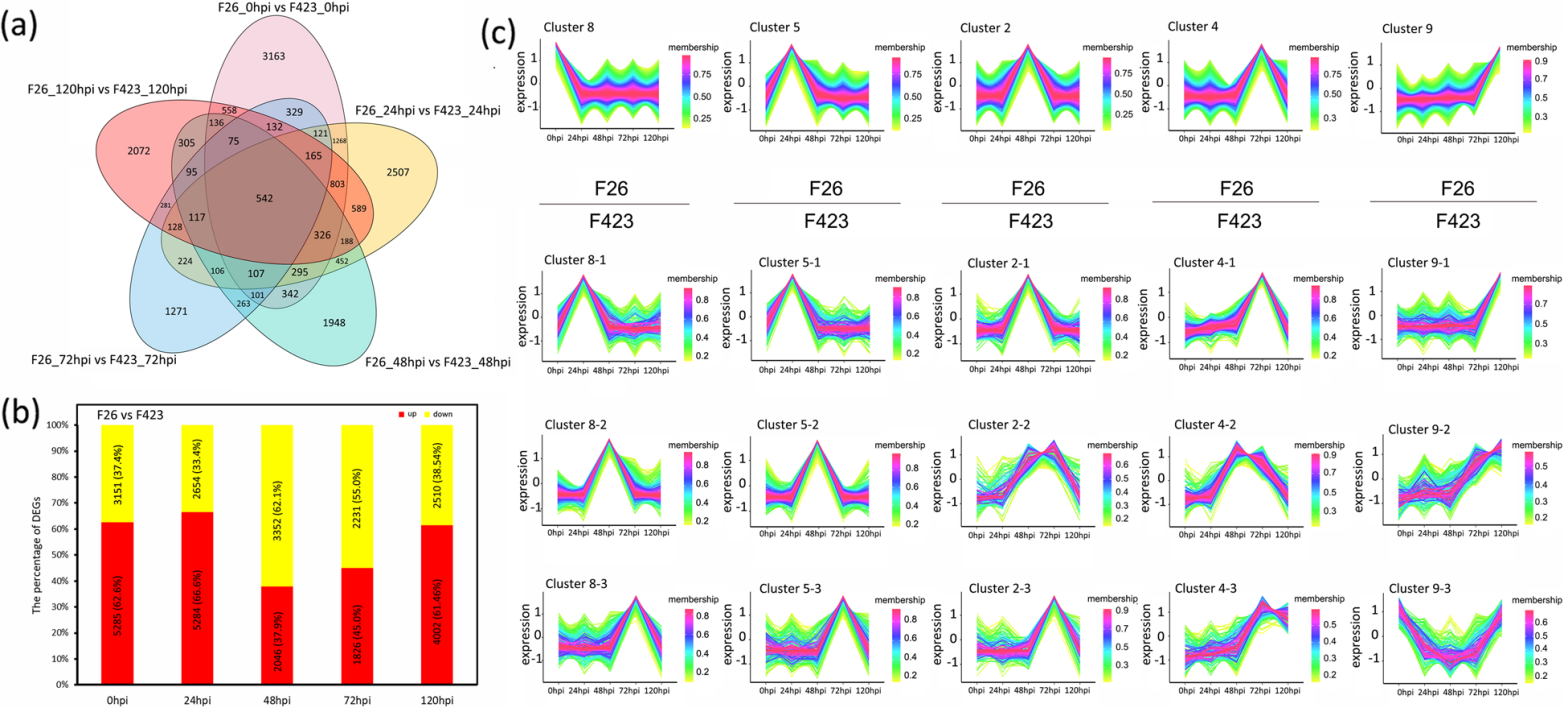
Comparative analysis of the response of F26 and F423 to *C. gloeosporioides* at the transcriptome level. **a** The Venn diagram presents the DEGs among five comparison groups. **b** The number and percentages of upregulated and downregulated DEGs among the five comparison groups. **c** Cluster analysis to identify the expression trend of time-series DEGs in F26 and 423

To compare the responses of F26 and F423 to *C. gloeosporioides*, we performed a comparative analysis of F26 vs. F423 at different stages of infection using the RNA-seq data. After mapping the clean reads of walnut (Table S1) to the reference genome, we identified 21,798 differentially expressed genes (DEGs) in F26 vs. F423 at five different stages. In the five comparisons, the number of DEGs was 8436 (5285 were upregulated) at 0 hpi, 7938 (5284 were upregulated) at 24 hpi, 5398 (2046 were upregulated) at 48 hpi, 4057 (1826 were upregulated) at 72 hpi, and 6512 (4002 were upregulated) at 120 hpi, respectively. A total of 542 DEGs were obtained at five different stages (Fig. 2a, b). The number of DEGs and upregulated DEGs of F26 vs. F423 in the early stage (0 hpi and 24 hpi) of infection was greater than that in the late stage (48 hpi, 72 hpi and 120 hpi). We subsequently performed a cluster analysis to identify the expression trend of the time-series DEGs in F26 and 423. Cluster analysis showed that the cluster was composed of 10 clusters (Figure S1), of which cluster 8 (0 hpi), cluster 5 (24 hpi), cluster 2 (48 hpi), cluster 4 (72 hpi), and cluster 9 (120 hpi) were stage specific in F26 in upper panels of Fig. 2c. Accordingly, the expression patterns of these genes stage specific in F26 were also analyzed in F423 in lower panels of Fig. 2c (Fig. 2c). Cluster 8 contained 2528 DEGs at 0 hpi, most of which exhibited delayed expressed in F423 (24 hpi, cluster 8–1; 48 hpi, cluster 8–2; 72 hpi, cluster 8–3). Similarly, some genes in cluster 5 (24 hpi) and cluster 2 (48 hpi) in F26 also showed delayed expression in F423 (48 hpi, cluster 5–2; 72 hpi, cluster 5–3; 72 hpi, cluster 2–3) (Fig. 2c). The expression patterns of many potential R genes were consistent with the above results. For example, G-type lectin S-receptor-like serine/threonine-protein kinase CES101 (*LOC109000970*) was highly expressed at 0 hpi in F26, but was expressed at 48 hpi and 72 hpi in F423 (Table S2). These results suggest that resistant fruits may display an earlier transcriptional response to *C. gloeosporioides* infection than susceptible fruits.

### GO and KEGG analysis of DEGs

**Fig. 3:**
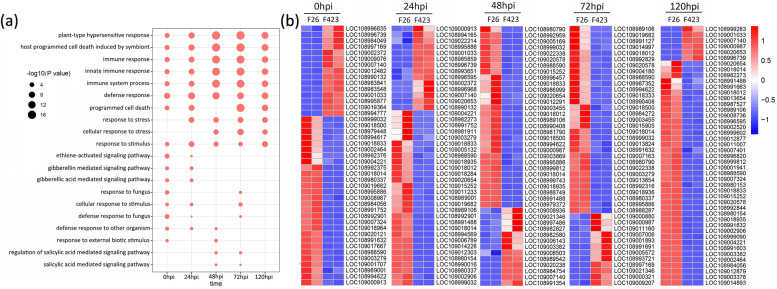
GO analysis and heatmap of DEGs. **a** GO analysis of DEGs at five infection stages in F26 vs F423. **b** Heatmap of DEGs in F26 and F423

To understand the function of DEGs in walnut resistance to *C. gloeosporioides*, we performed a functional enrichment analysis for GO terms in the DEGs at each time point for F26 vs. F423. The results showed that the DEGs at different stages of infection in F26 vs. F423 were enriched in many biological processes, cellular components, and molecular functions (Figure S2). We filtered the top 20 GO terms at each time point in which the DEGs were enriched (Table S3). Figure 3a depicts GO terms that were significantly enriched (*P*-adj ≤ 0.05) in at least two out of five time points. Multiple terms related to plant immunity, such as “plant-type hypersensitive response”, “host programmed cell death induced by symbiont”,“immune response”,“innate immune response”, “immune system process”, and “defense response and response to stimulus”, were enriched in the five periods (Fig. 3a). The expression of disease resistance protein At4g27190-like (LOC108988590) and putative disease resistance RPP13-like protein 2 (LOC109018014), related to the immune response, was upregulated in F26 at five periods (Fig. 3b). “Ethylene-activated signaling pathway”, “gibberellin mediated signaling pathway”, and “gibberellic acid mediated signaling pathway” were more evident at the early time point (Fig. 3a). *EDR1* (*LOC109003279*)*, ERF113* (*LOC108989001*)*, ABI4* (*LOC108991752*)*,* and *ESR2* (*LOC108992901*), related to these pathways, presented upregulated expression at 0 hpi and 24 hpi in F26 (Fig. 3b). However, “regulation of salicylic acid mediated signaling pathway” and “salicylic acid mediated signaling pathway” were enriched in 48 hpi and 72 hpi (Fig. 3a, b).

**Fig. 4:**
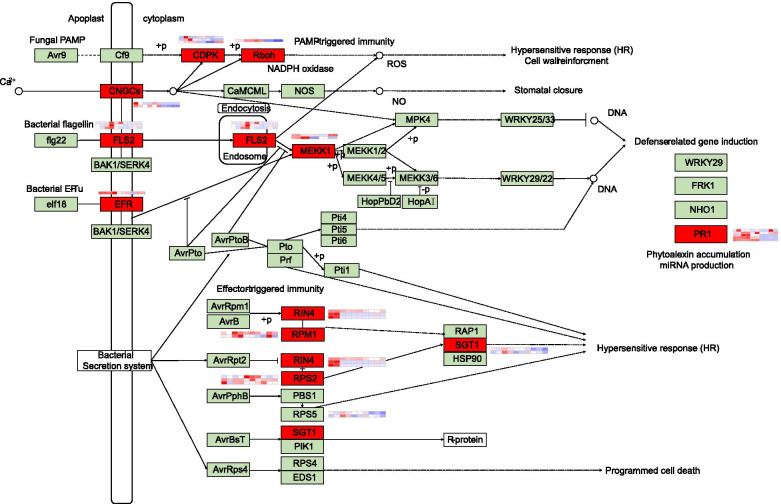
Expression network analysis of DEGs in the plant − pathogen interaction pathway by KEGG analysis. *CDPK, FLS2, EFR,* and *PR1* related to PAMP-triggered immunity were upregulated in F26 at 0 hpi and 24 hpi. *RIN4, PBS1, RPS2, RPM1,* and *SGT1* related to effector-triggered immunity were also found to be upregulated at early time points in F26

To study the host metabolic pathways altered during *C. gloeosporioides* progression, we performed a KEGG enrichment analysis. We found that many DEGs related to “phenylpropanoid biosynthesis”, “cutin suberine and wax biosynthesis”, “glucosinolate biosynthesis”, and “fatty acid elongation” were enriched at three time points at least (Figure S3). Given the enrichment of genes involved in “plant − pathogen interaction” across the five time points, a targeted analysis of the plant − pathogen interaction pathway was conducted. In the F26 vs. F423 comparison, DEGs related to PAMP-triggered immunity (e.g., *CDPK, FLS2, EFR, PR1*) were upregulated in F26 at 0 hpi and 24 hpi. DEGs related to effector-triggered immunity (e.g., *RIN4, PBS1, RPS2, RPM1, SGT1*) were also found to be upregulated at early time points in F26 (Fig. 4).

### Correlation network analysis with WGCNA

**Fig. 5:**
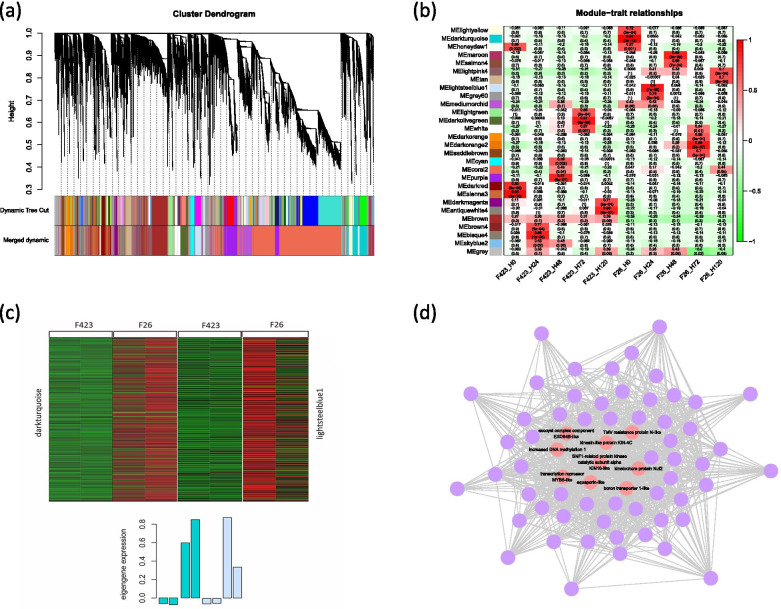
Correlation network analysis with WGCNA. **a** Hierarchical cluster tree presenting 27 modules of co-expressed mRNAs. **b** Heatmaps indicating the correlation of module eigengenes at different infection stages. The Pearson’s correlation coefficients of each module at various stages are provided and colored according to the score. **c** Heatmap indicating the eigengene expression in the ‘darkturquoise’ and ‘lightsteelblue1’ module in response to *C. gloeosporioides*. **d** Transcription expression gene networks by WGCNA. The hub genes and correlated genes are represented by orange and purple circles, respectively

To obtain the hub genes correlated with the resistance of walnut fruit to *C. gloeosporioides*, a weighted correlation network was constructed using the DEGs. In this study, we identified 27 distinct modules, shown in the dendrogram of Fig. 5a, where major tree branches define the modules (labeled with different colors in Fig. 5b). The relationships between modules and the resistance traits of the walnut fruit bracts were analyzed and the significantly correlated modules (|*r*|≥ 0.9) were found in the ‘darkturquoise’ (*r* = 0.97, *p* = 3e − 12) and ‘lightsteelblue1’ (*r* = 0.90, *p* = 7e − 08) modules (Fig. 5c). To further understand the mechanism of the resistance response, the enriched GO terms and KEGG pathways of the unigenes in the two modules were analyzed. In addition to the terms related to biological metabolism, the unigenes in the ‘darkturquoise’ module were mainly enriched in response to starvation (GO:0,009,267), response to nutrient levels (GO:0,031,669), and response to extracellular stimulus (GO:0,009,991) (Figure S7a), and the highly enriched terms of the ‘lightsteelblue1’ module were associated with induced systemic resistance (GO:0,009,864), jasmonic acid-mediated signaling pathway (GO:0,009,867), immune effector process (GO:0,002,252), and activation of innate immune response (GO:0,002,218) (Figures S4 and S5). The most significant entries in the KEGG analysis of the ‘darkturquoise’ and ‘lightsteelblue1’ modules were plant hormone signal transduction (ko04075) and starch and sucrose metabolism (ko00500), respectively (Figures S6 and S7).

WGCNA was also performed to construct transcription expression gene networks, in which each node represents a responsive gene, and the connecting lines between genes represent co-expression correlations. Hub genes which show the most connections in the network are important for regulating the whole network. In the ‘darkturquoise’ and ‘lightsteelblue1’ modules, nine hub genes were determined according to their weight value and connectivity in the modules, including aquaporin-like (*LOC108987002*), SNF1-related protein kinase catalytic subunit alpha KIN10-like (*LOC109006307*), kinetochore protein Nuf2 (*LOC109007030*), boron transporter 1-like (*LOC109004147*), transcription repressor MYB6-like (*LOC109006329*), increased DNA methylation 1(*LOC109012343*), kinesin-like protein KIN-4C (*LOC109013712*), exocyst complex component EXO84B-like (*LOC109003106*), and TMV resistance protein N-like (*LOC108996501*) (Fig. 5d). SNF1-related protein kinase catalytic subunit alpha KIN10-like and TMV resistance protein N-like are potential *R* genes in walnut. Details of the highlighted genes in the network are shown in Table S4.

### Quantitative real-time (qRT)-PCR analyses of selected genes

**Fig. 6:**
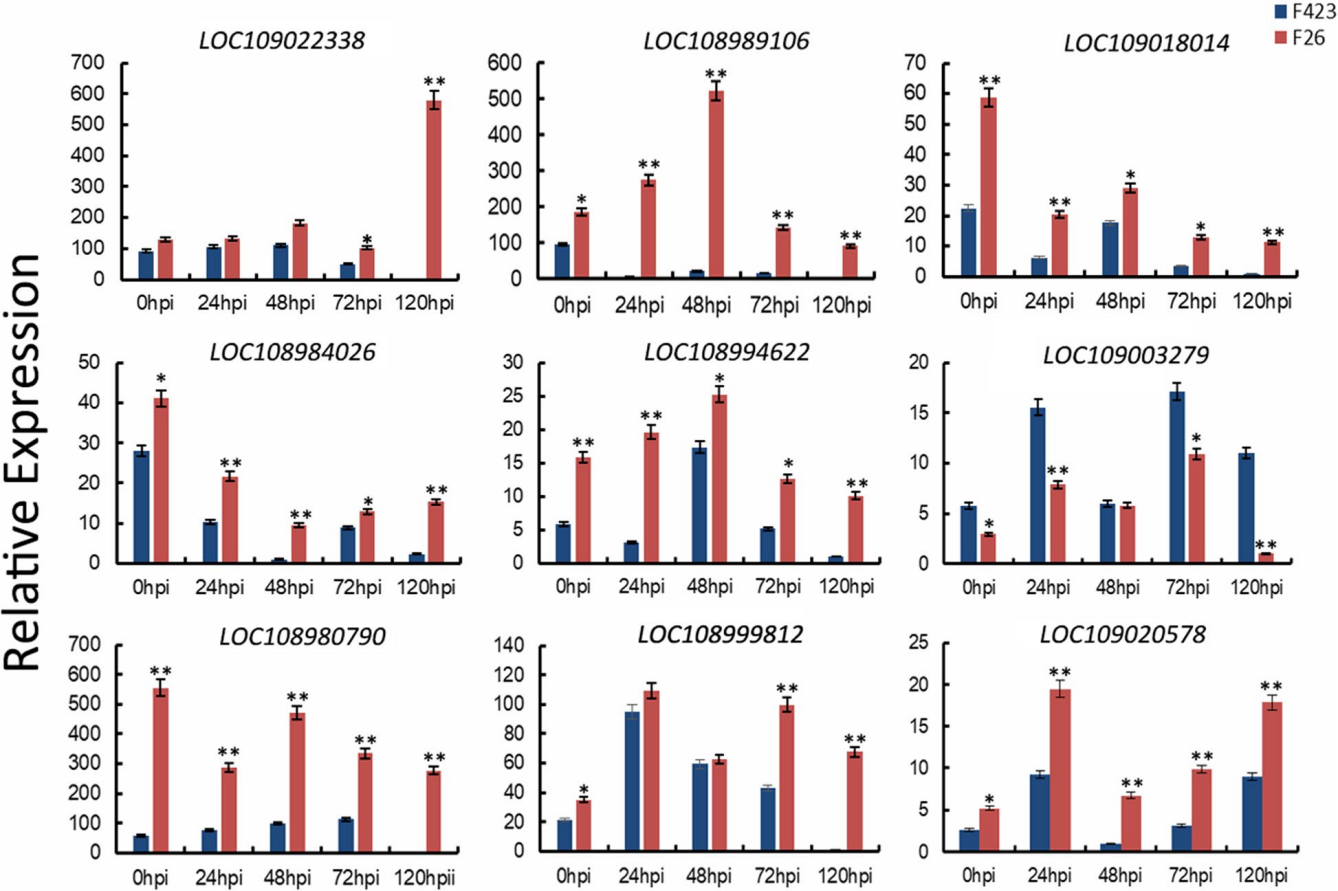
Validation of selected genes by qRT-PCR. Blue and red represent the F423 and F26 samples, respectively. Expression data were normalized against the data for the18S rRNA housekeeping gene and are presented as the mean ± standard error; *P < 0.05, **P < 0.01

To verify the expression profiles between F26 and F423 obtained by RNA-seq, we randomly selected nine genes for qRT-PCR analysis. The 18S rRNA gene was used as a housekeeping gene, and the transcript abundance of nine genes was normalized by comparison with the constitutive abundance of 18S rRNA (Fig. 6). The list of gene-specific primers used for qRT-PCR analysis is listed in Table S5. All the tested genes exhibited similar expression characteristics in the RNA-seq data (Table S6).

### Proteome analysis and functional enrichment analysis of DEPs

**Fig. 7:**
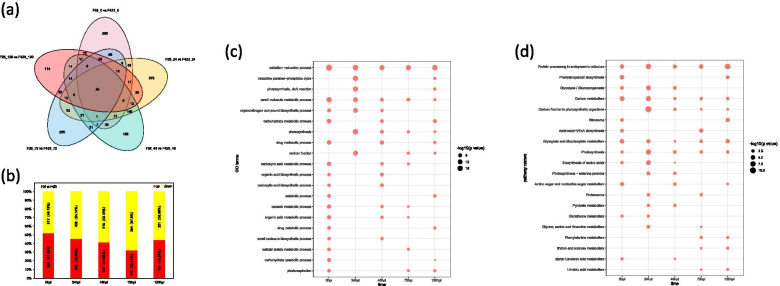
Proteome analysis and functional enrichment analysis of DEPs. **a** The Venn diagram presents the DEPs among the five comparison groups. **b** The numbers and percentages of upregulated and downregulated DEGs among the five comparison groups. **c** GO analysis of DEPs at five infection stages. **d** KEGG analysis of DEPs at five infection stages

The label-free quantitation analysis resulted in a total of 10,623,699 spectra, with 1,109,228 matching those of known peptides. Among them, 15,260 unique peptides were identified, and 5159 proteins were explored (Table S7). In the F26 vs. F423 comparison, a total of 648 DEPs (336 proteins upregulated and 312 proteins downregulated) at 0 hpi, 798 DEPs (366 proteins upregulated and 432 proteins downregulated) at 24 hpi, 545 DEPs (227 proteins upregulated and 318 proteins downregulated) at 48 hpi, 524 DEPs (170 proteins upregulated and 354 proteins downregulated) at 72 hpi, and 415 DEPs (184 proteins upregulated and 231 proteins downregulated) at 120 hpi were observed (Fig. 7a, b). The number of DEPs of F26 vs. F423 at 0 hpi and 24 hpi was greater than that in the late stage (48 hpi, 72 hpi and 120 hpi), which was consistent with the change trend of the number of DEGs.

GO categorization and KEGG pathway annotation were used to display the changes in DEPs annotated with functions. Similar to the functional enrichment analysis method of the DEGs, we filtered the top 20 GO terms at each time point in which the DEPs were enriched (Table S8). The GO terms in Fig. 7c were significantly enriched (*P*-adj ≤ 0.05) in at least two out of five time points. The DEPs participated in oxidation–reduction process (GO:0,055,114) and small molecule metabolic process (GO:0,044,281) during the five infection periods. Furthermore, many DEPs participated in photosynthesis (GO:0,015,979), carbohydrate metabolic process (GO:0,005,975), and carboxylic acid metabolic process (GO:0,019,752). Figure 7d shows the KEGG pathway enrichment analysis of DEPs at different points (*P* < 0.05) (Table S9). The most universally enriched KEGG pathways in all comparison groups included protein processing in endoplasmic reticulum (ko04141), carbon metabolism (ko01200), glyoxylate and dicarboxylate metabolism (ko00630), and photosynthesis (ko00195), which was similar to the enriched GO terms. For F26 vs. F423_0hpi, carbon metabolism was the most enriched pathway, while carbon fixation in photosynthetic organisms was the most enriched pathways for F26 vs. F423_24hpi, and protein processing in endoplasmic reticulum was the most enriched pathway for F26 vs. F423_48hpi, 72hpi, and 120hpi. These results suggested that plant secondary metabolic pathways, such as photosynthesis and carbon metabolism, might be related to walnut resistance to anthracnose.

### Correlation analysis between the transcriptome data and proteome data

**Fig. 8:**
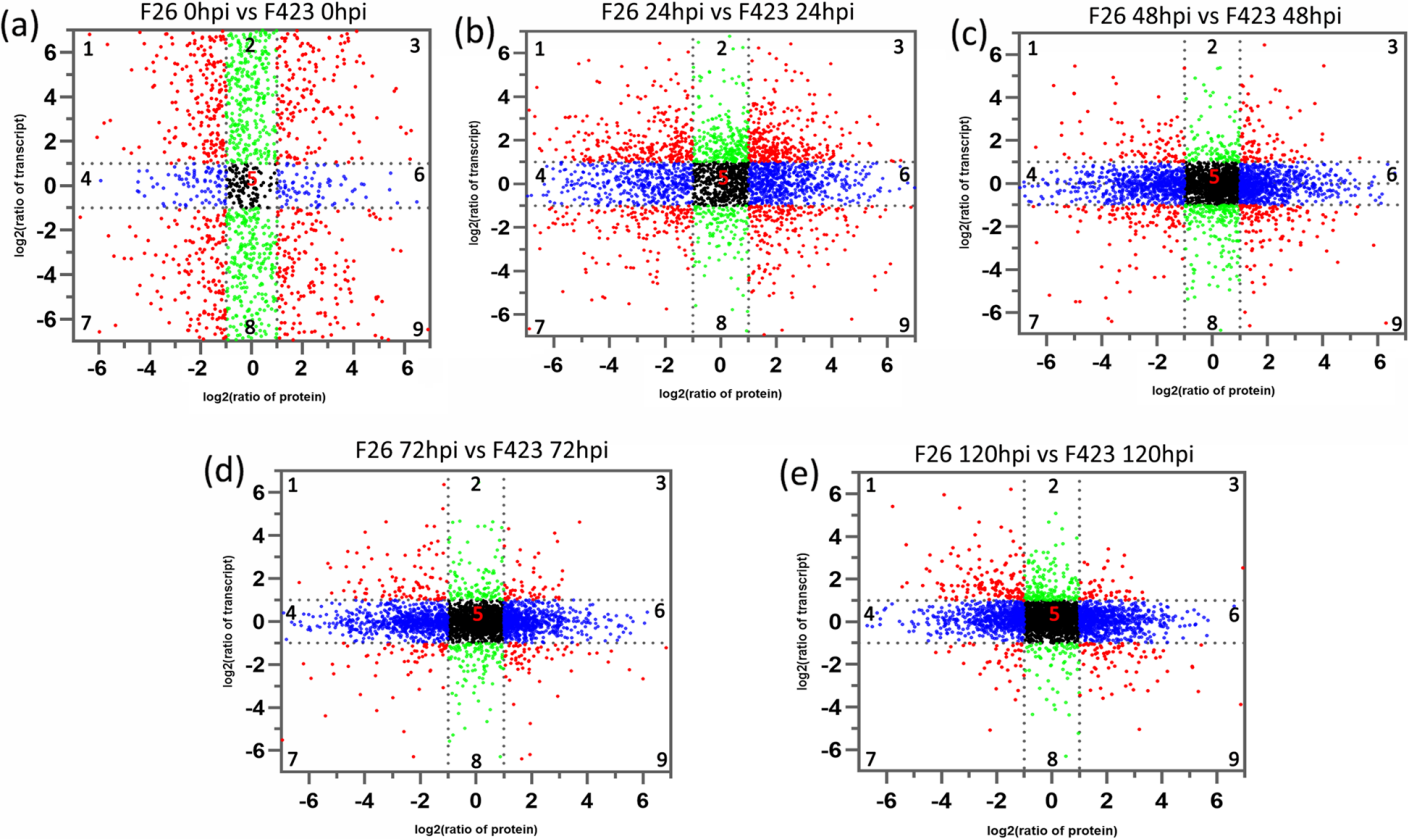
Division of genes into nine groups according to the log2 ratios of the protein species (x-axis) and transcripts (y-axis). Quadrants 1, 2 and 4 indicate that the protein abundance was lower than the RNA abundance. In quadrants 3 and 7, the RNAs correspond with the related proteins. Quadrant 5 shows that the proteins and RNAs were commonly expressed with no difference. Quadrants 6, 8 and 9 indicate that the protein abundance was higher than the RNA abundance. Comparison of changes in mRNA and cognate protein abundance. The relative change in abundance is shown on a log2 scale from samples among the five comparison groups. **a** F26_0hpi vs. F423_0hpi comparison; **b** F26_24hpi vs. F423_24hpi comparison; **c** F26_48hpi vs. F423_48hpi comparison; **d** F26_72hpi vs. F423_72hpi comparison; **e** F26_120hpi vs. F423_120hpi comparison

As the proteome data were obtained from the same walnut fruit samples used to produce the transcriptome data, we performed a correlation analysis of the transcriptomes and proteomes. Scatterplot analysis of the log2-transformed ratios was used to show the distribution of the corresponding mRNA: protein ratios. The genes detected in the proteome and transcriptome were divided into nine modules according to their expression patterns, establishing a nine- quadrant map (Fig. 8). There were 3644, 3031, 3028, 3034, and 3376 genes detected in both the proteome and the transcriptome at 0 hpi, 24 hpi, 48 hpi, 72 hpi, and 120 hpi in F26 vs. F423. Quadrants 1, 2 and 4 indicate that the protein abundance was lower than the RNA abundance. In quadrants 3 and 7, the RNAs correspond with the related proteins. Quadrant 5 shows that the proteins and RNAs were commonly expressed with no difference. Quadrants 6, 8 and 9 indicate that the protein abundance was higher than the RNA abundance. The number of genes whose mRNA and corresponding proteins exhibited consistent expression was 953 (26.15%), 511 (16.85%), 207 (6.83%), 121 (3.99%), and 92 (2.73%) at the five time points (Table S10). The differential expression consistency of the mRNA and corresponding protein was relatively higher in the early stage of infection. These results showed that genes were less regulated at the post-transcriptional or translational level in the early stage of infection. However, in the later stage, many genes were regulated at the post-transcriptional or translational level, such as miRNA and lncRNA-mediated protein degradation.

## Discussion

*Colletotrichum* species are fungal pathogens that devastate crop plants worldwide. Among these species, several lifestyles ranging from necrotrophy to hemibiotrophy have been identified [30]. In tomato, *C. gloeosporioides* breaches the fruit cuticle with a quiescent phase until fruit ripening signals a switch to necrotrophy [31]. In addition, most species complete the pathogenic process in a hemibiotrophic manner [32], typically penetrating the host tissues by melanized appressorium [33] and entering into the plant cells to start the abiotrophic phase before switching to a necrotrophic phase [34]. In this study, through scanning electron microscopy, aniline blue staining, and paraffin section observation, we observed that a penetration peg was formed at 24 hpi, entered into the intracellular space to start biotrophy at 48 hpi, and finally entered a necrotrophic stage with the formation of secondary and necrotrophic hyphae at 72 hpi. Therefore, *C. gloeosporioides* causing walnut fruit anthracnose completes its lifestyle transition in a hemibiotrophic manner, which is consistent with previous research results [25, 27].

The molecular mechanisms underlying these diverse infectious processes have been studied for several *Colletotrichum* species using transcriptomic and proteome analysis. Genome and transcriptome analyses of *C. higginsianum* infecting *Arabidopsis thaliana* and *C. graminicola* infecting maize found that pathogenicity-related genes are transcribed in successive waves that are linked to pathogenic transitions [27]. A large number of secondary metabolites and membrane transporters were identified during the infection of *C. falcatum* on sugarcane using RNA-seq technology [35]. A total of 304 proteins were identified from *C. lupini* during interaction with white lupin by mass spectrometry [30]. In the present study, based on the lifestyle transitions of *C. gloeosporioides*, the walnut fruit bracts tissues of F26 and F423 at 0 hpi, pathological tissues at 24 hpi, 48 hpi, and 72 hpi, and distal uninoculated tissues at 120 hpi were used for transcriptome and proteome analysis. A total of 21,798 DEGs and 1929 DEPs were identified in F26 vs. F423 using RNA-seq and label-free quantitation. Meanwhile, the correlation analysis of the transcriptome and proteome showed that the correlation between gene and protein expression was very weak in the late stage. Similar to multiple other studies [36, 37], the results showed that not all mRNA: protein ratios reflected the corresponding changes in transcription and protein levels [38], which was due to the possible occurrence of posttranscriptional regulation during the *C. gloeosporioides* response in the walnut fruit bracts.

The plant immune system consists of a two-tiered plant immune machinery: PAMP-triggered immunity (PTI) and ETI. PTI is a basic defense response activated by plasma member-anchored pattern recognition receptors (PRRs), which have been extensively studied in plants [3]. The PRR FLS2 can form a heterodimer complex with BAK1 to activate immune signaling upon binding a 22-amino acid epitope (flg22) conserved in bacterial flagellins [39, 40]. Another PRR EFR can recognize the conserved 18-aa epitope (elf18) of PAMP EF-Tu in bacteria to trigger immunity in Brassicaceae [6, 41]. The introduction of EFR in transgenic wheat, rice, tobacco, and tomato often confers bacterial resistance [6, 41]. In this study, *FLS2* and *EFR* related to PTI were upregulated in F26 at 0 hpi and 24 hpi in the F26 vs. F423 comparison, and multiple PRR pathways were activated simultaneously, which is likely to increase the robustness of the overall plant defense against pathogen infection [3, 42]. In addition, the number of DEGs and DEPs in F26 vs. F423 at the early stage was greater than that in the late stage. Additionally, compared with F26, many genes in F423 showed delayed expression patterns. For example, potential resistance genes (*R* genes) encoding walnut G-type lectin S-receptor-like serine/threonine-protein kinase CES101 (*LOC109000970*) were highly expressed at 0 hpi in F26, but were expressed at 48 hpi and 72 hpi in F423. These findings suggest that F26 may display an earlier and broader response to *C. gloeosporioides* than F423.

Pathogens can secret effector proteins to suppress host PTI, which in turn will elicit ETI [8, 43]. ETI is activated by intracellular immune receptors, most of which are nucleotide-binding leucine-rich repeat proteins (NLRs) encoded by *R genes* [44, 45]. Many *R genes* and their corresponding Avr effectors in pathogens have been identified. RPM1-INTERACTING PROTEIN 4 (*RIN4*) acts as a guard for the R protein NLRRPM1 in *Arabidopsis* and interacts with *Pseudomonas syringae* effectors AvrRpm1 and AvrB [46]. Similarly, the soybean GmRIN4b recognizes the *Pseudomonas syringae* effector AvrB as the guardee for the soybean RPG1-B [47]. RRS1/RPS4 in *Arabidopsis* and *RGA4/RGA5* in rice are well-studied NLR pairs that fit the integrated decoy model [48, 49]. In our study, *RIN4/RPM1* was enriched in the early stage (0 hpi and 24 hpi) in F26. Additionally, the expression levels of *RPS2* were higher in F26 when compared to F423 at 48 and 72 hpi. However, as more *R-genes* encoding detoxification enzymes and WAKs are cloned, it is becoming apparent that they confer resistance via a range of different mechanisms [45]. Nine hub genes related to disease resistance through WGCNA analysis were determined, and among them, SNF1-related protein kinase catalytic subunit alpha KIN10-like (*LOC109006307*) and TMV resistance protein N-like (*LOC108996501*) were potential *R* genes in walnut. KIN10 (SnRK1.1 and SnRK1α1) encodes a kinase α-subunit of Snf1-related kinase1 (SnRK1), which can phosphorylate key metabolic enzymes to immediately influence their activity [45]. The N gene of tobacco (*Nicotiana tabacum*) is a typical *R gene* engendering the localization of tobacco mosaic virus (TMV) infection and the elicitation of a hypersensitive necrotic response [50]. In addition, three hub potential *R* genes (G-type lectin S-receptor-like serine/threonine-protein kinase LECRK1, *LOC108979712*; probably inactive leucine-rich repeat receptor-like protein kinase At2g25790, *LOC109009481*; and TMV resistance protein N-like, *LOC108988638*) obtained in the study of Feng et al. [29] also present in the WGCNA module in this study. Therefore, the roles of these potential *R genes* in walnut resistance to anthracnose were worth further study.

ETI does not work as a strategy against necrotrophs, as it is only effective against biotrophs and hemibiotrophs in their early stages [50]. After the initial infection, SAR can be activated, which protects distant parts in the plant [50]. SA has been established as a major hormone that triggers a response against pathogens with a biotrophic lifestyle. Generally, the mediation of SA is fine-tuned by other hormones, such as ethylene (ET) and gibberellins (GAs) [51]. ET is a hydrocarbon gas that is involved in plant immunity. The *Pseudomonas* effectors AvrPto and AvrPtoB trigger ET production in tomato, which might work indirectly through the downregulation of SA signaling by AvrPtoB [52, 53]. As pathogens targeting GA signaling interact with the more important defense pathways, SA responses against biotrophic pathogens are increased in *della* mutants [54]. In this study, DEGs related to “ethylene-activated signaling pathway,” “gibberellin mediated signaling pathway,” and “gibberellic acid mediated signaling pathway” were enriched at 0 hpi and 24 hpi. *EDR1* (LOC109003279)*, ERF113* (LOC108989001)*, ABI4* (LOC108991752)*,* and *ESR2* (LOC108992901), which are related to these pathways, presented upregulated expression in F26. In addition, “regulation of salicylic acid mediated signaling pathway” and “salicylic acid mediated signaling pathway” were enriched at 48 hpi and 72 hpi. These results suggested that ET and GAs were involved in the resistance of walnut to anthracnose at the early stage, and SA-mediated SAR might be formed at 48 hpi and 72 hpi. In addition, photosynthesis and secondary metabolic pathways have been reported to protect the plant under biotic and abiotic stresses [55, 56]. The GO and KEGG analysis of DEPs in F26 vs. F423 showed that the carbon metabolism and photosynthesis-related pathways were highly induced at different stages. These results indicate that photosynthesis-related genes may play an important role in walnut resistance to *C. gloeosporioides*.

## Conclusions

Collectively, in this study, comparative transcriptome and proteome analyses were simultaneously conducted to investigate the mechanisms of walnut resistance to *C. gloeosporioides* between F26 and F423. We showed that the lifestyle transitions of *C. gloeosporioides* in infected walnut fruits, and large amounts of DEGs and DEPs, were detected in F26 vs. F423 at different stages. Comparative analysis of F26 vs. F423 at the transcriptome level showed that F26 may display an earlier and broader response to *C. gloeosporioides* than F423. Two modules and nine hub genes significantly related to the resistance to *C. gloeosporioides* were identified through WGCNA. Meanwhile, numerous DEGs were mainly related to the immune response, plant hormone signal transduction, and secondary metabolites, and the DEPs were involved in carbon metabolism and photosynthesis. Although many additional studies are needed to elucidate their functions, our present results improve our understanding of the molecular mechanisms underlying the regulatory networks of walnut resistance to *C. gloeosporioides,* identifying many candidate genes and proteins that could be applied to the breeding of walnut disease resistance.

## Methods

### Plant materials and strains

The fruits of the B26 clone (i.e., F26) and 4–23 clone (i.e., F423) were used as experimental materials. The scions of the walnut seedling tree B26 were provided by the walnut specialized farmers’ cooperative of Dongliugang village, Baishi Town, Wenshang County, Shandong Province, China (35°46′56.2″N, 116°40′30.8″E). The 4–23 walnut tree was from F1 progeny of an intraspecific cross between walnut cultivar ‘Yuan Lin’ (susceptible to anthracnose) × ‘Qing Lin’ (resistant to anthracnose), which we carried out in 2002. The plant materials were conserved by patch budding onto walnut seedling rootstock at the Forestry Experimental Station of Shandong Agricultural University, Tai’an, Shandong Province, China (36°10′ 19.2″N, 117°09′ 1.3″E) in late May 2009. According to our evaluation results of anthracnose resistance, as previously described [24], in 2015–2017, we found that the B26 clone was highly resistant to anthracnose in the fruit bract, and the 4–23 clone was highly susceptible to anthracnose in the fruit bract. The strain ‘m9’ of *C. gloeosporioides* (GenBank ID: GU597322) was used in the inoculations.

### Inoculation on walnut fruits

The *C. gloeosporioides* isolates were gown on potato dextrose agar (PDA, potato 200 g·L^−1^, glucose 20 g·L^−1^, agar 17 g·L^−1^) medium for 5 d before producing spores. To prepare conidial suspensions, the colonies were washed with sterile distilled water containing 0.05% (v/v) Tween 80, passed through a filter (40 – 100 μm porosity), quantified using a hemocytometer, and diluted with sterile distilled water to 106 conidia·mL^−1^ (0.001% final concentration of Tween 80). Punch inoculation of detached walnut fruits was performed as described in Li et al. [57].

### Anatomical changes of walnut tissue during *C. gloeosporioides* infection

#### Observation of the germination process of *C. gloeosporioides* spores

The spare spore suspension was diluted with 1% dextrose aqueous solution (pH = 6.8) to about 25 – 30 conidia under the 10 × visual field of the optical microscope and then placed on a concave glass slide and cultured in an HSX-150 temperature and humidity incubator (temperature 28 °C, humidity 95%). The suspension was observed and photographed with an ECLIPSE 90i optical microscope (Nikon, Japan) every 6 h.

#### Observation of aniline blue staining

The inoculated walnut tissue was first decolorized (0.15% trichloroacetic acid (w ·V^−1^) was dissolved in 3:l (V·V^−1^) mixed solution of ethanol and chloroform for 14 h) and then stained with aniline blue for 2 h. The walnut tissue infected by *C. gloeosporioides* conidia was observed and photographed using an ECLIPSE 90i optical microscope every 6 h.

#### Observation of paraffin section

The infected sample was fixed with FAA fixative (10 mL formalin, 3 mL acetic acid, 87 mL 50% ethanol) for 8 – 24 h, dehydrated by gradient ethanol, made transparent with xylene, and then embedded in wax. We used an automatic rotary microtome (Leica RM2255, Germany) to slice, deparaffinize, and clear the sections with xylene, rehydrate them in an ethanol gradient, and then stain them with aniline saffron. Finally, the sections were sealed and dried. The paraffin sections were observed and photographed using an ECLIPSE 90i optical microscope.

#### Scanning electron microscope observation

The infested samples were double fixed with 4% glutaraldehyde and osmium tetroxide overnight and then washed with 0.01 mol·L^−1^ pH 6.8 PBS (phosphate buffered saline) 6 – 8 times. After washing, the samples were dehydrated by gradient ethanol, dehydrated in 100% acetone twice, and then replaced with isoamyl acetate twice. After the sample was dried using a K850 CO_2_ critical point dryer (EMITECH, UK) at room temperature, it was glued and coated. A JSM-6610LV scanning electron microscope was used for observation and photography (JEOL, Japan) at 15 kV.

### Walnut tissue samples

The anatomical observation of the infection process indicated that the completed lifestyle stage transitions of *C. gloeosporioides* were at 0, 24, 48 and 72 hpi. Hence, the inoculated site at 0, 24, 48, and 72 hpi and distal uninoculated tissue at 120 hpi were collected to perform transcriptome and proteome analyses. The samples were collected, immediately frozen in liquid nitrogen, and then stored at − 80 °C until analysis. Three biological replicated were collected at each infection time.

### RNA extraction, library construction, and RNA-seq

Total RNA was extracted using a Thermo GeneJET Plant RNA Purification Mini Kit (Thermo Fisher Scientific Inc, USA) with a DNase treatment according to the manufacturer’s protocols. The integrity and quality of the total RNA was checked using a NanoDrop 2000 (Thermo Fisher Scientific Inc, USA) and Qubit 2.0 RNA Broad Range Assay (Invitrogen, USA). The quality of the RNA was examined using an Agilent Bioanalyzer RNA Nano chip Bioanalyzer (Agilent Technologies) to obtain an RNA Integrity number (RIN) over 7. After poly (A) selection and rRNA depletion, the mRNA was purified and fragmented using fragmentation buffer (Thermo Fisher Scientific Inc, USA). The RNA fragments underwent reverse transcription using random hexamer-primers. This was followed by second-strand cDNA synthesis with DNA polymerase I, dNTPs, and RNase H. After end repair, A-tailing, and index ligation, the products were purified with a QiaQuick PCR extraction kit and amplified to create the final cDNA libraries. The synthesized cDNA was quantified using a Qubit 2.0 DNA Broad Range Assay (Invitrogen, USA). The RNA libraries of tested samples were sequenced on an Illumina HiSeq 4000 Platform (Illumina, USA) to generate 2 × 150 bp paired-end sequencing reads.

### Alignment to the genome and transcript assembly

The quality of the sequences was assessed in fastq format using FASTQC (version 0.10.1) (www.bioinformatics.bbsrc.ac.uk/projects/fastqc.). Adaptors and low-quality bases were trimmed from the sequences using Trimmomatic. Reads with a phred quality (Q > 30) were used for further alignment and assembly. The clean reads obtained after filtration were aligned to the NCBI *Juglans regia* genome sequence (https://www.ncbi.nlm.nih.gov/genome/?term=Juglans%20regia) using HISAT (v 2.0.5). The aligned reads for each sample were assembled into transcripts using Stringtie (v 1.3.1). The StringTie assembly results were combined by StringTie–Merge, and gene expression was calculated using StringTie's own script prepDE.py.

### Protein extraction and digestion

Walnut fruit bracts at different infection stages were used for protein extraction, which was performed using cold acetone extraction, as described previously. Samples were ground to powder in liquid nitrogen and then dissolved in 10 mL of lysis buffer (7 M urea, 2 M thiourea, 0.1% (w/v) CHAPS, 30 mM Tris, 2% protease inhibitor, pH 8.0), followed by incubation on ice for 15 min and centrifugation at 14,000 rpm for 30 min at 4 °C. The supernatant was then transferred to a new tube for standby. Protein concentrations were measured with the Bradford Regent Kit (Sigma, Poole, Dorset, UK) according to the manufacturer’s protocols.

The obtained protein was reduced with 5 mM dithiothreitol for 1 h at 37 °C, alkylated with 15 mM iodoacetamide for 1 h at room temperature, and digested with trypsin (porcine sequencing grade modified trypsin; Promega) by applying a 1:20 (w/w) trypsin: protein ratio and incubating at 37 °C overnight. The obtained peptides from digestion were dried in vacuum.

### Liquid chromatography tandem-mass spectrometry (LC–MS/MS) analysis

Label-free LC–MS/MS analysis was performed on an ultra-nanoflow high-performance liquid chromatography (HPLC) system (EASY-nLC™ 1000, Thermo Fisher Scientific, Sunnyvale, CA, USA) coupled online via a nano-electrospray ion source to a Nano HPLC Q Exactive System (Thermo Fisher Scientific, Sunnyvale, CA, USA). Tryptic digests (2 μg/μL) were diluted 1:10 with aqueous 0.1% formic acid (FA), and 5 μL was loaded on a trap column Acclaim PepMap100 (100 μm × 2 cm; C18, 5 μm, 100 Å) and then eluted over the Thermo Scientific EASY-Spray column (75 μm × 15 cm, C18, 3 μm, 100 Å) at 350 nL/min. Both columns were packed with Jupiter Proteo resin (Phenomenex, Torrance, CA, USA). Peptides were eluted following the gradient: 0 –5 min (4% Buffer B), 5–40 min (15%-25% Buffer B), 40–65 min (25%-35% Buffer B), 65–70 min (35%-95% Buffer B), 70–82 min (95% Buffer B), 82–85 min (95%-4% Buffer B), and 85–90 min (4% Buffer B) [Buffer B (100% acetonitrile, 0.1% formic acid), Buffer A (0.1% formic acid)]. Mass spectra were retrieved by Xcalibur (version 2.2, Thermo Fisher Scientific, Bremen, Germany). The mass spectrometer was operated in positive ion mode at unit resolution. Each fraction was analyzed in triplicate in technical replicates. The mass spectrometer was operated under full scan mode, the scan range was 350 – 1800 m/z, the spray voltage was 2.0 kv, the capillary temperature was 250 °C, and the injection time was set 60 ms. The top-ten most intense ions were selected for collision induced dissociation (CID) using a normalized collision energy of 30%. Data were collected over a broad mass to charge (*m/z*) precursor ion selection scan range of 350–1800 m*/z* with an isolation window of 3 m*/z*. Dynamic exclusion was used to minimize redundant selection of peptides previously selected for CID with the following settings: repeat count = 1, repeat duration = 30 s, exclusion list size = 500, exclusion duration = 90 s, and expiration S/N threshold = 3.

### Protein identification and label-free quantification

The raw data files were processed and quantified using MaxQuant software (version 1.6.1.0) and searched against the NCBI *Juglans regia* proteome database (https://www.ncbi.nlm.nih.gov/genome/?term=Juglans+regia). The peptide precursor mass tolerance was set at 10 ppm, and the MS/MS tolerance was set at 0.8 Da. Search criteria included carbamido methylation of cysteine (+ 57.0214) as a fixed modification and oxidation of methionine (+ 15.9949). For the downstream biological analysis of highly multivariate quantitative protein abundance data generated using mass-spectrometry-based analysis, the Perseus software platform (http://www.perseus-framework.org) was used for data normalization. Hierarchical clustering was done with the gplots package. The Short Time-series Expression Miner (STEM) was introduced to the analysis of short time series protein expression data. Peptide ion abundance in the three replicates was used to calculate the expression level of each protein. The abundance level of a protein was quantified with the sum of ion peak intensity of the most abundant precursors of the tryptic peptides. Calculations of the protein *P*-value (one-way ANOVA) were made on the sum of the normalized abundances across all runs.

### Analysis of differential expression patterns

Genes and proteins differentially expressed between F26 and F423 at five infection stages were analyzed with DESeq2 (version 1.22.1) and Ballgown (2.14.0). After assessing the significance of any differences, the genes and proteins with a *P*-value ≤ 0.05 and a |log2foldchange|≥ 1 were designated as differentially expressed genes and proteins.

### Time-course expression clustering analysis

TCseq (v1.4.0) was used to analyze the time-series expression data of all the DEGS and DEPs. The default parameters (k = 15, algo = cmeans) were set, and 15 clusters were generated.

### Weighted gene correlation network analysis

According to the method of Langfelder and Horvath [58], WGCNA (v1.63) (http://lab.genetics.ucla.edu/horvath/CoexpressionNetwork/) was used to construct a weighted gene/protein coexpression network in walnut fruit at 0, 24, 48, 72, and 120 hpi. Firstly, the weight (power) of more than 80% modules suitable for scale-free topology and low mean connectivity was taken as the soft threshold *β*-value. To generate a number of clusters, modules were defined after eliminating or combining branches. The co-expression module dynamic shear tree parameters were determined as described by Gerttula [59]. The minimum number of genes was set to 30, the split sensitivity (deep Split) was set to 2, and the other settings were the default parameters of the software. The Module Eigengenes (ME) of each module were calculated, and the module correlation according to the ME was calculated, and the modules with a correlation coefficient greater than 0.90 were identified. Correlations among the modules with the resistance traits of different infection stages were calculated, following which the correlation matrix between the modules and genes was calculated. According to the correlation between the trait and the ME and the *P*-value, the modules related to the trait were mined, and the module with the highest correlation coefficient and the smallest *P*-value was selected as the most relevant module for the trait. The co-expression networks of hub-genes in highly correlated modules were generated with the Cytoscape software (version 3.7.1).

### Functional enrichment analysis

All annotated genes obtained from transcriptome sequencing were used as background genes for GO and KEGG analysis. The topGO (v 2.26.0) software was used to search the GO database (http://geneontology.org/) for the genes/proteins analyzed, and the GO term that satisfied *P* ≤ 0.05 was defined as a significantly enriched GO term. KOBAS (v 2.0) software was used to search the KEGG database (http://www.genome.jp/kegg/) to determine the significantly enriched KEGG metabolic pathway (*P* ≤ 0.05). The method of Benjamini–Hochberg was used to correct p-value.

### Gene expression data validation by qRT-PCR

The primers used for qRT-PCR were designed with Beacon Designer 7 software and were synthesized by Sangon Biotech (Shanghai, China; Table S4). The qRT-PCR was performed using the TransStart Tip Green qPCR SuperMix (Transgen) and the CFX Connect Real-Time System (Bio-Rad). The amplification conditions were 95 °C for 3 min, followed by 40 cycles of 95 °C for 10 s and 60 °C for 30 s. Samples for qRT-PCR were run in three biological replicates. The results were normalized using the 2^−ΔΔCt^ method (Software IQ5 2.0) to report relative expression [60]. For normalization of gene expression, 18S rRNA gene was used as an internal standard.

### Correlation analysis of transcriptome and proteome data

In order to understand the correlation between transcriptome and proteome data at different stages of infection, according to the relative difference multiples between the transcriptome and the proteome, the default screening threshold was set to 2 times that of the proteins and the transcript group. After log2 transformation, GraphPad Prism 8 (v8.3.0.538) was used to draw a nine- quadrant map of transcripts and proteins of differential expression.

## Supplementary Information

**Additional file 1: Figure S1**. Time course sequencing data analysis of F26 under *C. gloeosporioides* for expression genes.

**Additional file 2: Figure S2**. The GO terms of DEGs in F26 vs F423 at each infection stage. (a) F26_0hpi vs F423_0hpi comparison. (b) F26_24hpi vs F423_24hpi comparison. (c) F26_48hpi vs F423_48hpi comparison. (d) F26_72hpi vs F423_72hpi comparison. (e) F26_120hpi vs F423_120hpi comparison.

**Additional file 3: Figure S3**. The significant KEGG pathways of DEGs in F26 vs F423 at each infection stage.

**Additional file 4: Figure S4**. The significant GO terms of genes in darkturquoise module.

**Additional file 5: Figure S5**. The significant GO terms of genes in lightsteelblue1 module.

**Additional file 6: Figure S6**. The significant KEGG pathways of genes in darkturquoise module.

**Additional file 7: Figure S7**. The significant KEGG pathways of genes in lightsteelblue1 module.

**Additional file 8: Table S1**. The summary of RNA-seq data.

**Additional file 9: Table S2**. Time course sequencing data analysis of F26 under *C. gloeosporioides* for expression genes.

**Additional file 10: Table S3**. The list of GO terms for DEGs in F26 vs F423 at each stage.

**Additional file 11: Table S4**. The hub-genes of darkturquoise and lightsteelblue1 module.

**Additional file 12: Table S5**. Sequences of quantitative real time PCR primers.

**Additional file 13: Table S6**. The expression of all the genes in the RNA-seq data.

**Additional file 14: Table S7**. The Protein quantitative information.

**Additional file 15: Table S8**. The list of GO terms for DEPs in F26 vs F423 at each stage.

**Additional file 16: Table S9**. The list of KEGG pathways for DEPs in F26 vs F423 at each stage.

**Additional file 17: Table S10**. The genes detected in the proteome and transcriptome in F26 vs F423 at each stage.

## Data Availability

The raw sequencing data were deposited in NCBI Sequence Read Archive under the accession number GSE147083 (https://www.ncbi.nlm.nih.gov/geo/query/acc.cgi?acc=GSE147083).

## Abbreviations

DEGs: Differentially expressed genes
DEPs: Differentially expressed proteins
PTI: Pathogen-associated molecular pattern (PAMP)-triggered immunity
ETI: Effector-triggered immunity
WGCNA: Weighted Gene Coexpression Network Analysis
Hpi: Hours post inoculation
GO: Gene Ontology
KEGG: Kyoto Encyclopedia of Genes and Genomes
ROS: Reactive oxygen species
SA: Salicylic acid
JA: Jasmonic acid
HR: Hypersensitive response
MAPK: Mitogen-activated protein kinase
SAR: Systemic acquired resistance
PRRs: Pattern-recognition receptors
PAMPs: Pathogen-associated molecular patterns
NLRs: Nucleotide-binding, leucine-rich repeat proteins
PR: Pathogenesis-related
GRNs: Gene regulatory networks
CDPK: Calcium dependent protein kinase
FLS: Flavonol synthase
R-genes: Resistance genes
RIN4: RPM1-INTERACTING PROTEIN 4
TMV: Tobacco mosaic virus

## References

[1] Deng Y, Ning Y, Yang DL, Zhai K, He Z (2020). Molecular basis of disease resistance and perspectives on breeding strategies for resistance improvement in crops. Mol Plant.

[2] Spoel SH, Dong X (2012). How do plants achieve immunity? Defence without specialized immune cells. Nat Rev Immunol.

[3] Saijo Y, Loo EP, Yasuda S (2018). Pattern recognition receptors and signaling in plant-microbe interactions. Plant J.

[4] Boutrot F, Zipfel C (2017). Function, discovery, and exploitation of plant pattern recognition receptors for broad-spectrum disease resistance. Annu Rev Phytopathol.

[5] Miya A, Albert P, Shinya T, Desaki Y, Ichimura K, Shirasu K, Narusaka Y, Kawakami N, Kaku H, Shibuya N (2007). CERK1, a LysM receptor kinase, is essential for chitin elicitor signaling in Arabidopsis. Proc Natl Acad Sci U S A.

[6] Zipfel C, Kunze G, Chinchilla D, Aniard AC, Jones J, Boller T, Felix G (2006). Perception of the bacterial PAMP EF-Tu by the receptor EFR restricts Agrobacterium-mediated transformation. Cell.

[7] Zipfel C, Robatzek S, Navarro L, Oakeley EJ, Jones J, Felix G, Boller T (2004). Bacterial disease resistance in Arabidopsis through flagellin perception. Nature.

[8] Cui H, Tsuda K, Parker JE (2014). Effector-triggered immunity: from pathogen perception to robust defense. Annu Rev Plant Biol.

[9] Dodds PN, Rathjen JP (2010). Plant immunity: towards an integrated view of plant-pathogen interactions. Nat Rev Genet.

[10] Horsefield S, Burdett H, Zhang X, Manik MK, Shi Y, Chen J, Qi T, Gilley J, Lai JS, Rank MX (2019). NAD+ cleavage activity by animal and plant TIR domains in cell death pathways. Science.

[11] Wang J, Hu M, Wang J, Qi J, Han Z, Wang G, Qi Y, Wang HW, Zhou JM, Chai J (2019). Reconstitution and structure of a plant NLR resistosome conferring immunity. Science.

[12] Fu ZQ, Dong X (2013). Systemic acquired resistance: turning local infection into global defense. Annu Rev Plant Biol.

[13] Zhang H, Fu Y, Guo H, Zhang L, Wang C, Song W, Yan Z, Wang Y, Ji W (2019). Transcriptome and proteome-based network analysis reveals a model of gene activation in wheat resistance to stripe rust. Int J Mol Sci.

[14] Reddy A, Marquez Y, Kalyna M, Barta A (2013). Complexity of the alternative splicing landscape in plants. Plant Cell.

[15] Janke C, Bulinski JC (2011). Post-translational regulation of the microtubule cytoskeleton: mechanisms and functions. Nat Rev Mol Cell Biol.

[16] Moreno-Risueno MA, Busch W, Benfey PN (2010). Omics meet networks - using systems approaches to infer regulatory networks in plants. Curr Opin Plant Biol.

[17] Zhang XQ, Bai L, Sun HB, Yang C, Cai BY (2020). Transcriptomic and proteomic analysis revealed the effect of funneliformis mosseae in soybean roots differential expression genes and proteins. J Proteome Res.

[18] Walley JW, Sartor RC, Shen Z, Schmitz RJ, Wu KJ, Urich MA, Nery JR, Smith LG, Schnable JC, Ecker JR (2016). Integration of omic networks in a developmental atlas of maize. Science.

[19] Martínez García PJCM (2016). The walnut (Juglans regia) genome sequence reveals diversity in genes coding for the biosynthesis of non-structural polyphenols. Plant J.

[20] Stevens KA, Woeste K, Chakraborty S, Crepeau MW, Langley CH (2018). Genomic variation among and within six juglans species. G3 Genes Genom Genet.

[21] Bernard A, Marrano A, Donkpegan A, Brown PJ, Dirlewanger E (2020). Association and linkage mapping to unravel genetic architecture of phenological traits and lateral bearing in Persian walnut (Juglans regia L.). BMC Genomics.

[22] Marranob A, Sideli GM, Leslie CA, Cheng H, Neale DB (2019). Deciphering of the genetic control of phenology, yield, and pellicle color in Persian walnut (Juglans regia L.). Front Plant Sci.

[23] Marrano A, Arcía PM, Bianco L, Sideli GM, Neale DB. A new genomic tool for walnut (Juglans regia L.): development and validation of the high-density Axiom™ J. regia 700K SNP genotyping array. Plant Biotechnol J 2018;17(39):1027-1036. 10.1111/pbi.13034 PMC6523593 30515952

[24] Zhu Y, Yin Y, Yang K, Li J, Sang Y, Long H, Shu F (2015). Construction of a high-density genetic map using specific length amplified fragment markers and identification of a quantitative trait locus for anthracnose resistance in walnut (Juglans regia L.). BMC Genomics.

[25] Gan P, Ikeda K, Da I, Narusaka M, O’Connell RJ, Takano Y, Kubo Y, Shirasu K (2013). Comparative genomic and transcriptomic analyses reveal the hemibiotrophic stage shift of Colletotrichum fungi. New Phytol.

[26] Mcdowell JM (2013). Genomic and transcriptomic insights into lifestyle transitions of a hemi-biotrophic fungal pathogen. New Phytol.

[27] O’Connell RJTMHS (2012). Lifestyle transitions in plant pathogenic Colletotrichum fungi deciphered by genome and transcriptome analyses. Nat Genet.

[28] An H, Yang K (2014). Resistance gene analogs in walnut (Juglans regia) conferring resistance to Colletotrichum gloeosporioides. Euphytica.

[29] Feng S, Fang H, Liu X, Dong Y, Yang KQ (2021). Genome-wide identification and characterization of long non-coding RNAs conferring resistance to Colletotrichum gloeosporioides in walnut (Juglans regia). BMC Genomics.

[30] Dubrulle G, Picot A, Madec S, Corre E, Pensec F (2020). Deciphering the infectious process of Colletotrichum lupini in lupin through transcriptomic and proteomic analysis. Microorganisms.

[31] Alkan N, Friedlander G, Ment D, Prusky D, Fluhr R (2015). Simultaneous transcriptome analysis of Colletotrichum gloeosporioides and tomato fruit pathosystem reveals novel fungal pathogenicity and fruit defense strategies. New Phytol.

[32] Dean R, Kan J, Pretorius ZA, Kosack KEH, Pietro AD, Spanu PD, Rudd JJ, Dickman M, Kahmann R, Ellis J (2012). The top 10 fungal pathogens in molecular plant pathology. Mol Plant Pathol.

[33] Kubo Y, Takano Y (2013). Dynamics of infection-related morphogenesis and pathogenesis in colletotrichum orbiculare. J Gen Plant Pathol.

[34] Gomes S, Prieto P, Martins-Lopes P, Carvalho T, Martin A, Guedes-Pinto H (2009). Development of colletotrichum acutatum on tolerant and susceptible Olea europaea L. cultivars: a microscopic analysis. Mycopathologia.

[35] Prasanth N, Viswanathan R, Malathi P, Sundar AR, Krishna N (2017). Unraveling the genetic complexities in gene set of sugarcane red rot pathogen colletotrichum falcatum through transcriptomic approach. Sugar Tech.

[36] Meng X, Liu S, Dong T, Xu T, Zhu M (2020). Comparative transcriptome and proteome analysis of salt-tolerant and salt-sensitive sweet potato and overexpression of IbNAC7 confers salt tolerance in arabidopsis. Front Plant Sci.

[37] Zhang L, Huang X, He C, Zhang QY, Zou X, Ke D, Gao Q (2018). Novel fungal pathogenicity and leaf defense strategies are revealed by simultaneous transcriptome analysis of colletotrichum fructicola and strawberry infected by this fungus. Front Plant Sci.

[38] Saad H, Ranadip P (2013). Integrated analysis of transcriptomic and proteomic data. Curr Genomics.

[39] Daire X, Fernandez O, Adrian M, Zipfel C, Dorey S, Poinssot B, Trda L, Kelloniemi J, Heloir MC, Boutrot F (2014). The grapevine flagellin receptor VvFLS2 differentially recognizesflagellin-derived epitopes from the endophytic growthpromotingbacterium Burkholderia phytofirmans and plantpathogenic bacteria. New Phytol.

[40] Takai R, Isogai A, Takayama S, Che FS (2009). Analysis of flagellin perception mediated by flg22 receptor OsFLS2 in rice. Mol Plant Microbe Interact.

[41] Boller T, He SY (2009). Innate immunity in plants: an arms race between pattern recognition receptors in plants and effectors in microbial pathogens. Science.

[42] Couto D, Zipfel C (2016). Regulation of pattern recognition receptor signalling in plants. Nat Rev Immunol.

[43] Boller T, Felix G (2009). A renaissance of elicitors: perception of microbe-associated molecular patterns and danger signals by pattern-recognition receptors. Annu Rev Plant Biol.

[44] Li W, Deng Y, Ning Y, He Z, Wang GL (2020). Exploiting broad-spectrum disease resistance in crops: from molecular dissection to breeding. Annu Rev Plant Biol.

[45] Nelson R, Wiesner-Hanks T, Wisser R, Balint-Kurti P (2018). Navigating complexity to breed disease-resistant crops. Nat Rev Genet.

[46] Mackey D, Iii B, Wiig A, Dangl JL (2002). RIN4 interacts with Pseudomonas syringae type III effector molecules and is required for RPM1-mediated resistance in Arabidopsis. Cell.

[47] Selote D, Robin GP, Kachroo A (2013). GmRIN4 protein family members function nonredundantly in soybean race-specific resistance against Pseudomonas syringae. New Phytol.

[48] Baggs E, Dagdas G, Krasileva KV (2017). NLR diversity, helpers and integrated domains: making sense of the NLR IDentity. Curr Opin Plant Biol.

[49] Sarris P, Duxbury Z, Huh S, Ma Y, Segonzac C, Sklenar J, Derbyshire P, Cevik V, Rallapalli G, Saucet S (2015). A plant immune receptor detects pathogen effectors that target WRKY transcription factors. Cell.

[50] Levy M (2004). Tobacco mosaic virus regulates the expression of its own resistance gene N. Plant Physiol.

[51] Bürger M, Chory J (2019). Stressed out about hormones: how plants orchestrate immunity. Cell Host Microbe.

[52] Chen H, Chen J, Li M, Chang M, Xu K, Shang Z, Zhao Y, Palmer I, Zhang Y, Mcgill J. A bacterial type III effector targets the master regulator of salicylic acid signaling, NPR1, to subvert plant immunity. SSRN Electron J 2017:S831720477. 10.1016/j.chom.2017.10.019 29174403

[53] Cohn JR, Martin GB (2010). Pseudomonas syringae pv. tomato type III effectors AvrPto and AvrPtoB promote ethylene-dependent cell death in tomato. Plant J.

[54] Navarro L, Bari R, Achard P, Lisón P, Nemri A, Harberd NP, Jones J (2008). DELLAs control plant immune responses by modulating the balance of jasmonic acid and salicylic acid signaling. Curr Biol.

[55] Bernacki MJ, Czarnocka W, Witoń D, Rusaczonek A, Szechyńska-Hebda M, Ślesak I, Dąbrowska-Bronk J, Karpiński S (2018). Enhanced disease susceptibility 1 (EDS1) affects development, photosynthesis, and hormonal homeostasis in hybrid aspen (Populus tremula L.×P. tremuloides) - sciencedirect. J Plant Physiol.

[56] Bartwal A, Mall R, Lohani P, Guru SK, Arora S (2013). Role of secondary metabolites and brassinosteroids in plant defense against environmental stresses. J Plant Growth Regul.

[57] Li W, Zhu Z, Chern M, Yin J, Yang C, Ran L, Cheng M, He M, Wang K, Wang J (2017). A natural allele of a transcription factor in rice confers broad-spectrum blast resistance. Cell.

[58] Langfelder P, Horvath S (2008). WGCNA: an R package for weighted correlation network analysis. BMC Bioinformatics.

[59] Gerttula S, Zinkgraf M, Muday GK, Lewis DR, Groover A (2015). Transcriptional and hormonal regulation of gravitropism of woody stems in populus. Plant Cell.

[60] Wang N, Liu W, Yu L, Guo Z, Chen X (2020). Heat shock factor A8a modulates flavonoid synthesis and drought tolerance. Plant Physiol.

