# Supplementary figures and images for: Transcriptome and proteome analysis of walnut (Juglans regia L.) fruit in response to infection by Colletotrichum gloeosporioides
Source: BMC Plant Biol. 2021 May 31;21:249. doi: 10.1186/s12870-021-03042-1 (PMC8166054; doi:10.1186/s12870-021-03042-1)

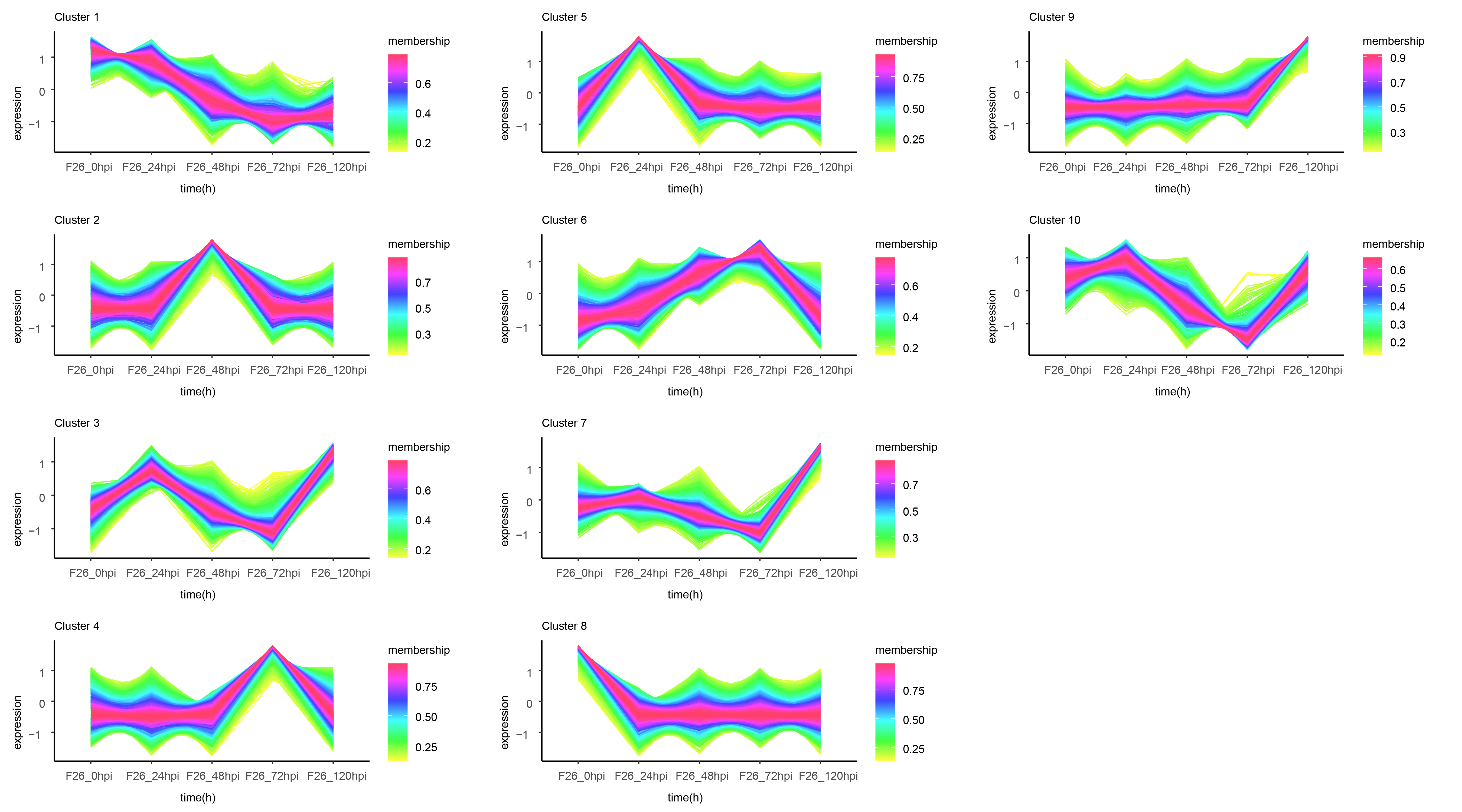

Supplement: Supplementary file 1 — Additional file 1: Figure S1. Time course sequencing data analysis of F26 under C. gloeosporioides for expression genes. [file 12870_2021_3042_MOESM1_ESM.tif]

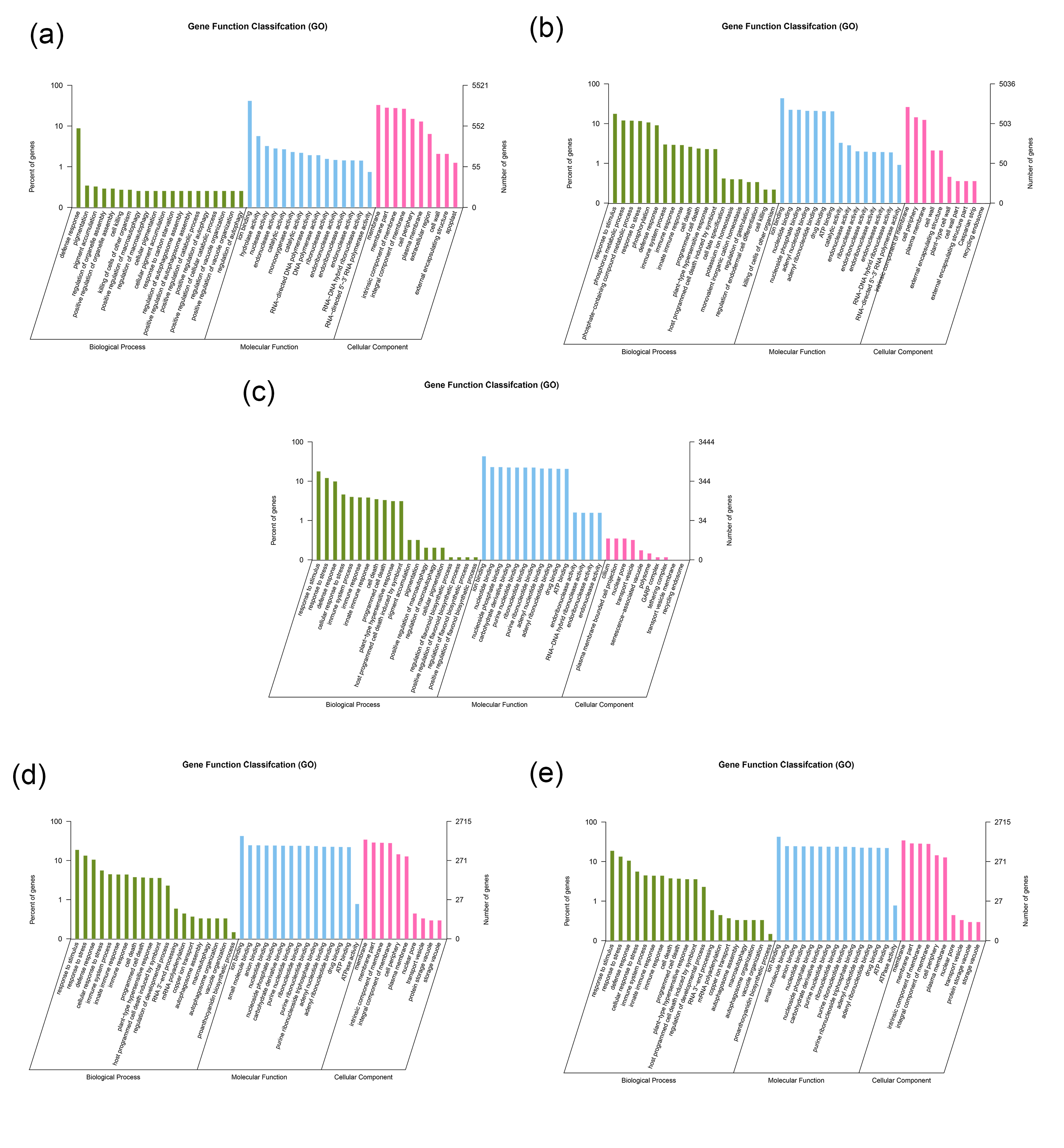

Supplement: Supplementary file 2 — Additional file 2: Figure S2. The GO terms of DEGs in F26 vs F423 at each infection stage. (a) F26_0hpi vs F423_0hpi comparison. (b) F26_24hpi vs F423_24hpi comparison. (c) F26_48hpi vs F423_48hpi comparison. (d) F26_72hpi vs F423_72hpi comparison. (e) F26_120hpi vs F423_120hpi comparison. [file 12870_2021_3042_MOESM2_ESM.tif]

pathways

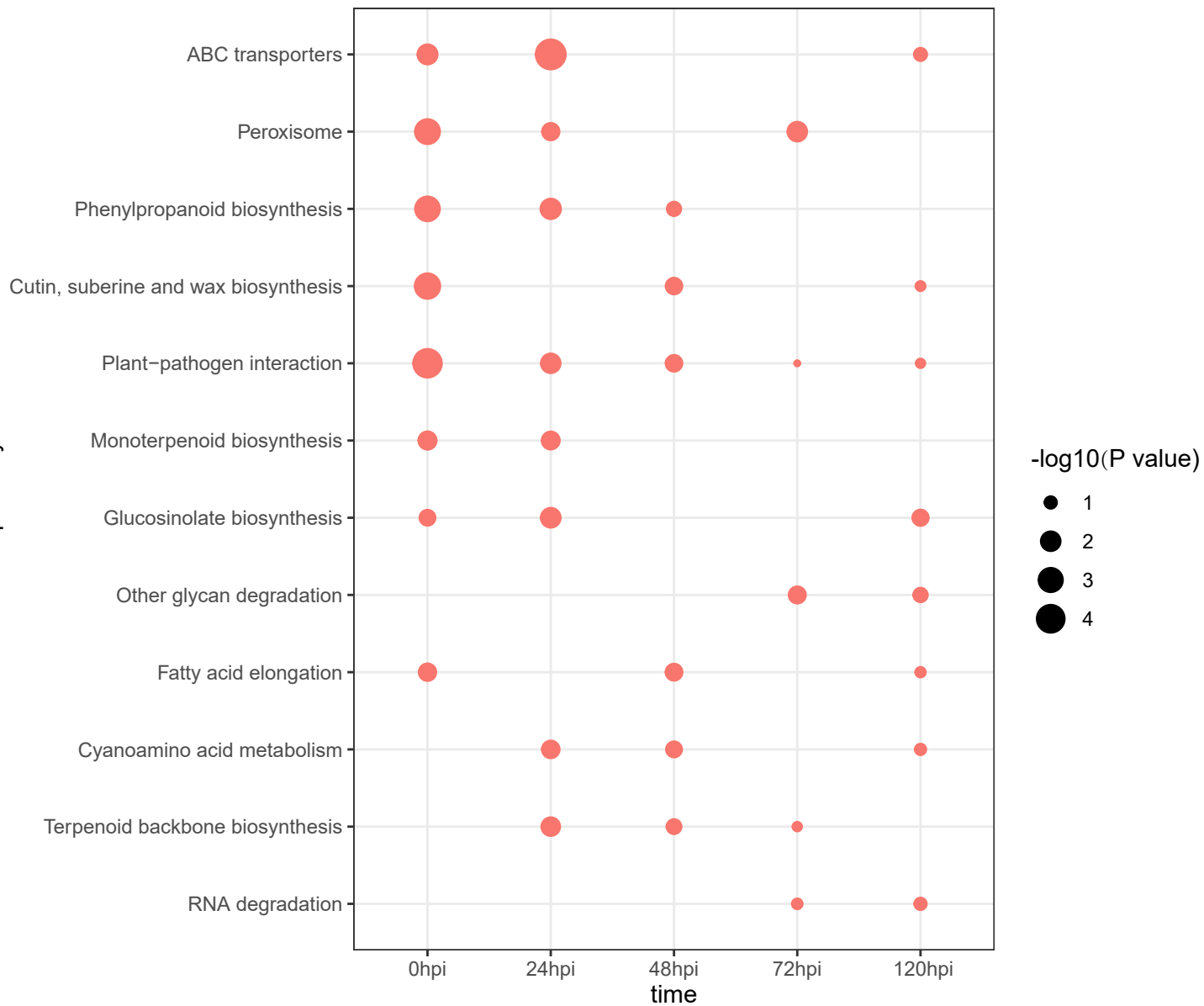

Supplement: Supplementary file 3 — Additional file 3: Figure S3. The significant KEGG pathways of DEGs in F26 vs F423 at each infection stage. [file 12870_2021_3042_MOESM3_ESM.pdf]

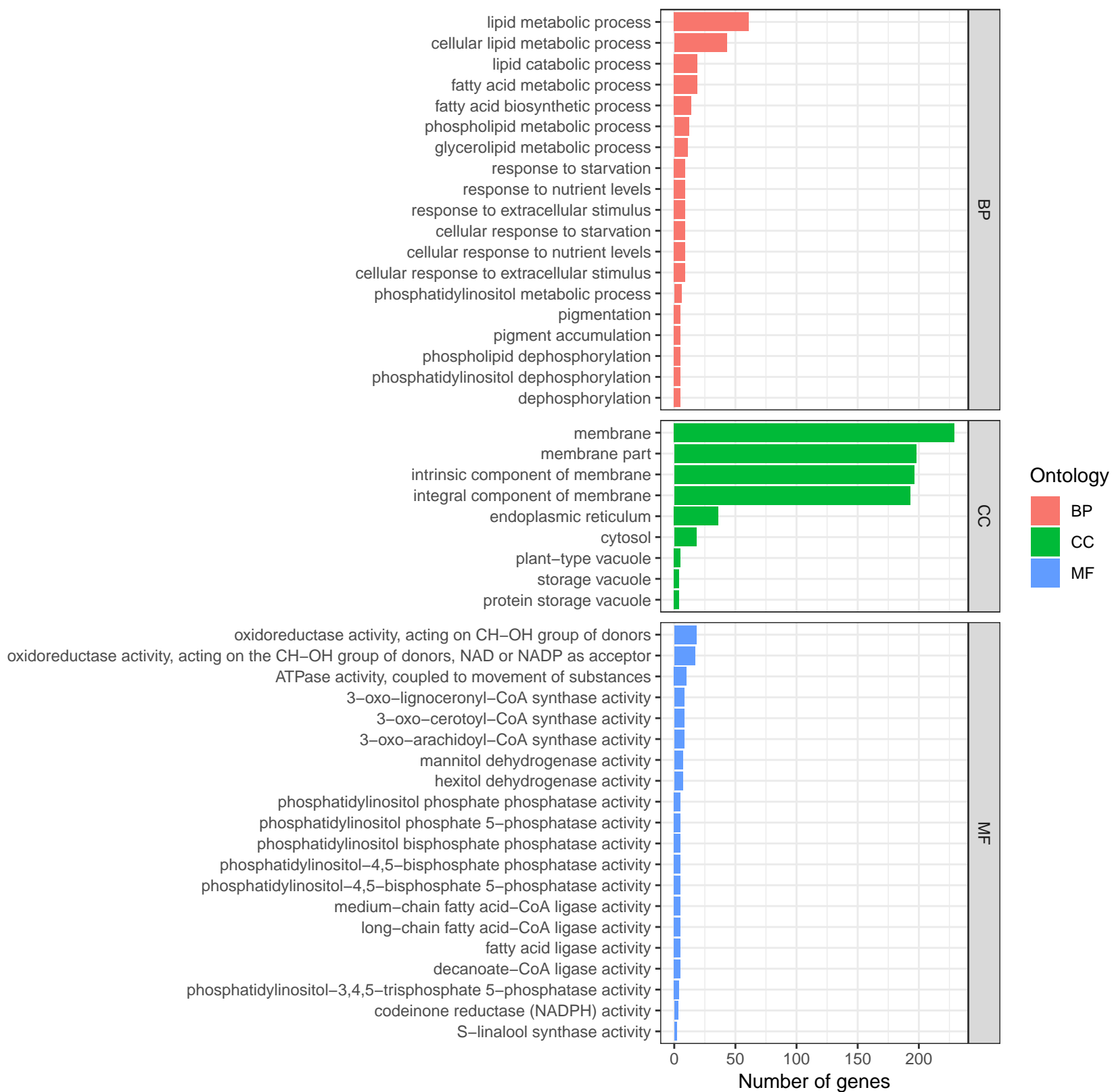

Supplement: Supplementary file 4 — Additional file 4: Figure S4. The significant GO terms of genes in darkturquoise module. [file 12870_2021_3042_MOESM4_ESM.pdf]

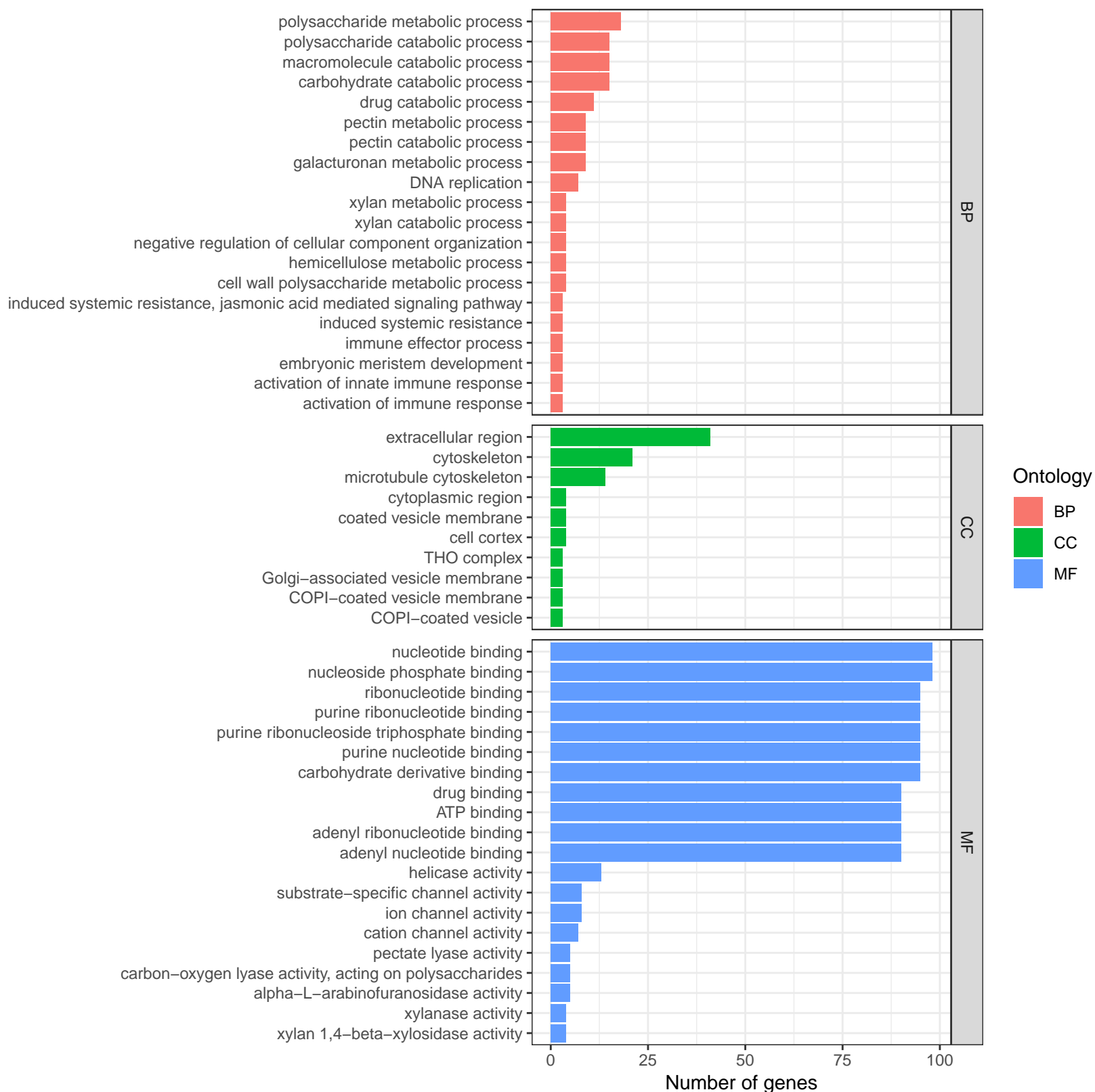

Supplement: Supplementary file 5 — Additional file 5: Figure S5. The significant GO terms of genes in lightsteelblue1 module. [file 12870_2021_3042_MOESM5_ESM.pdf]

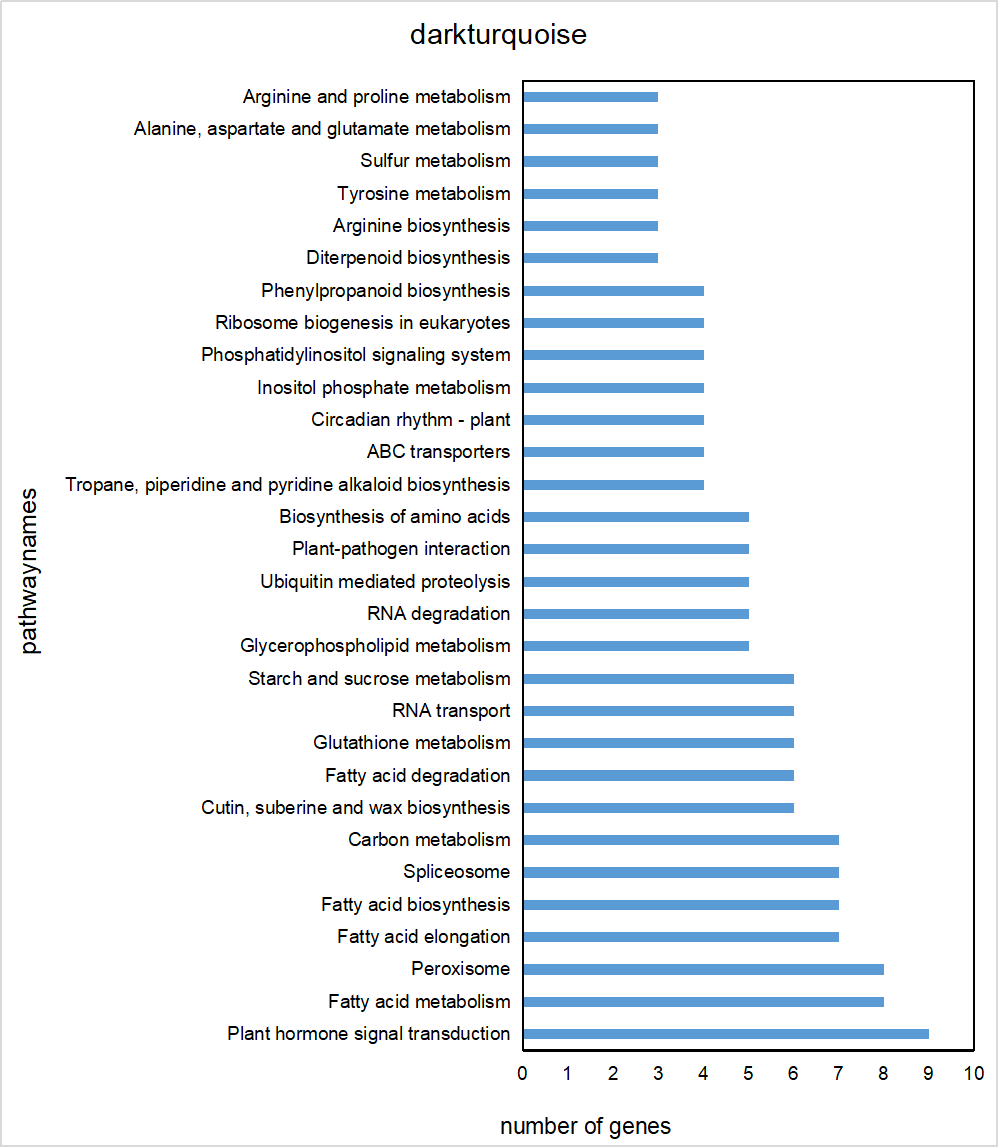

Supplement: Supplementary file 6 — Additional file 6: Figure S6. The significant KEGG pathways of genes in darkturquoise module. [file 12870_2021_3042_MOESM6_ESM.tif]

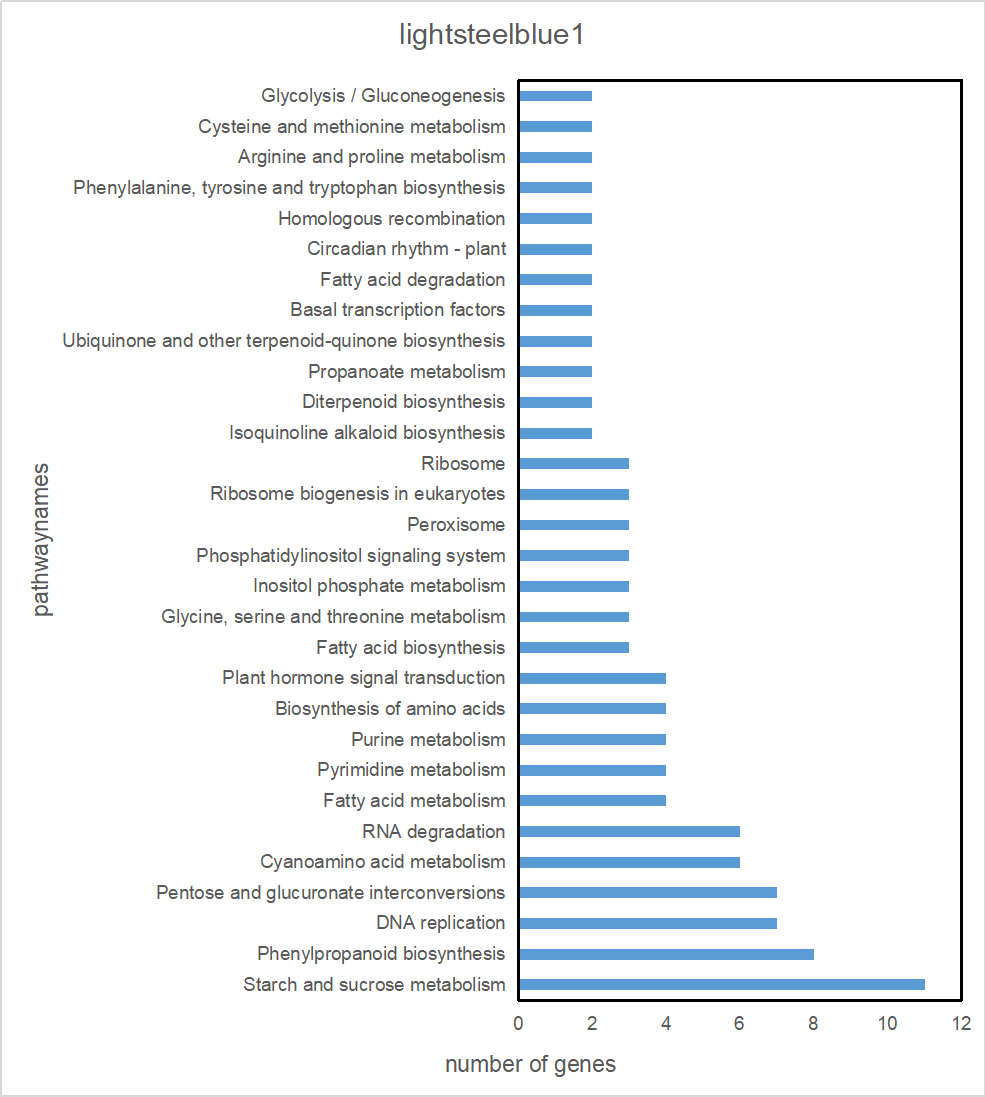

Supplement: Supplementary file 7 — Additional file 7: Figure S7. The significant KEGG pathways of genes in lightsteelblue1 module. [file 12870_2021_3042_MOESM7_ESM.tif]
